# Chestnut Tannin Improves Growth Performance and Intestinal Health of Broilers Challenged with Necrotic Enteritis via the cGAS-STING-Ferroptosis Pathway

**DOI:** 10.3390/ani16040686

**Published:** 2026-02-22

**Authors:** Genrui Zhang, Fandi Tang, Yang Wang, Huawei Liu

**Affiliations:** College of Animal Science and Technology, Qingdao Agricultural University, Qingdao 266109, China; 20232209017@stu.qau.edu.cn (G.Z.); 20232109034@stu.qau.edu.cn (F.T.); yangwang@qau.edu.cn (Y.W.)

**Keywords:** broiler, cGAS-STING, chestnut tannin, ferroptosis, intestinal health

## Abstract

This study aimed to investigate the impacts of chestnut tannin (CT) on growth performance, immune response, and intestinal health of broilers challenged with necrotic enteritis (NE) through the cyclic GMP-AMP synthase (cGAS)-stimulator of interferon genes (STING)-ferroptosis pathway. A total of 240 one-day-old male Cobb 500 broilers (44.54 ± 0.51 g) were randomly divided into four groups, including a Control group, NE group, 500 mg/kg CT group (L-CT), and 1000 mg/kg CT group (H-CT), with six replicates per group and ten broilers per replicate. Sporulated coccidia oocysts on day 14 and *Clostridium perfringens* solution from days 19 to 21 were given to all broilers except the Control group through oral administration to establish the NE infection model. The results demonstrated that dietary supplementation with CT improved (*p* < 0.05) growth performance, intestinal morphology, and intestinal mucosal barrier function of broilers challenged with NE. CT supplementation decreased (*p* < 0.05) interleukin (IL)-1β, IL-6, type I interferon, interferon-γ, and tumor necrosis factor-α concentrations and increased (*p* < 0.05) IL-10 concentration in the jejunal mucosa. Furthermore, CT supplementation decreased (*p* < 0.05) Fe^2+^ concentration, malondialdehyde concentration, mitochondrial DNA level, and mitochondrial reactive oxygen species level in the jejunal mucosa. Broilers under NE challenge had upregulated (*p* < 0.05) jejunal protein expression of cGAS, STING, phospho-TANK-binding kinase 1, phospho-interferon regulatory factor 7, phospho-nuclear factor kappa B, ferroptosis suppressor protein 1, prostaglandin-endoperoxide synthase 2, acyl-CoA synthetase long-chain family member 4, WD repeat domain phosphoinositide-interacting protein 2, nuclear receptor co activator factor 4 and autophagy related protein 5 and downregulated (*p* < 0.05) glutathione peroxidase 4, ferritin heavy chain 1, ferritin light chain and ferroportin 1 compared with the Control group, while the supplementation of CT reversed these effects. In conclusion, CT improved intestinal inflammatory damage of broilers challenged with NE by inhibiting the cGAS-STING-ferroptosis pathway, which was more effective at a dose of 1000 mg/kg in this study.

## Simple Summary

Necrotic enteritis (NE) caused by *Clostridium perfringens* leads to diarrhea and intestinal inflammatory damage, seriously impairing the growth performance and health status of broilers. The excessive dependence on antibiotics for the treatment of NE in poultry production has led to the continuous increase in bacterial resistance and drug residues in products. Thus, there is an urgent need to find substitutes for treating NE. Chestnut tannin (CT) is a natural polyphenolic compound extracted from chestnut wood, which has strong antibacterial and anti-inflammatory functions. However, the mechanism by which CT plays a role in broilers challenged with NE remains for further study. In this study, dietary supplementation with CT improved the growth performance, intestinal morphology, intestinal barrier function, and immune response of NE-challenged broilers by inhibiting the cGAS-STING-ferroptosis pathway.

## 1. Introduction

As a bacterial disease, necrotic enteritis (NE) affects the intestinal tract of poultry, mainly induced by *Clostridium perfringens* (*C. perfringens*) and is associated with susceptibility factors including coccidiosis and fish meal [1,2]. *C. perfringens* produces NE toxin B-like (NetB) [3], which could induce decreased nutrient utilization [4], imbalanced intestinal microbiota [5], and impaired gut barrier [6], resulting in decreased growth performance, diarrhea, and increased mortality in broilers. The excessive dependence on antibiotics for the treatment of NE in poultry production has led to the continuous increase in bacterial resistance and drug residues in products, which result in a decrease in poultry disease resistance, as well as food safety issues [7]. Therefore, the quest for feasible natural alternatives has attracted widespread attention.

Chestnut tannin (CT) is a natural polyphenolic compound extracted from chestnut wood, mainly consisting of ellagitannins and gallotannins [8], which belongs to the category of hydrolysable tannins [9] and has functions including anti-inflammatory, antioxidant, antibacterial, and immune regulation [10]. Previous studies suggested that CT inhibited *C. perfringens* activities in vitro [11], reduced the expression of inflammatory cytokines in *C. perfringens*-infected intestinal epithelial cells [12], and improved intestinal lesion scores and morphological structure in broilers challenged with *C. perfringens* [13]. However, the mechanism by which CT plays a role in broilers challenged with NE remains to be further studied.

In the treatment of inflammatory diseases, the cyclic GMP-AMP synthase (cGAS)-stimulator of interferon genes (STING) pathway and ferroptosis play crucial roles [14]. Activated through the release of mitochondrial DNA (mtDNA) [15], the cGAS-STING pathway triggers various regulated cell deaths, including pyroptosis, necroptosis, and ferroptosis [16], and subsequently induces the massive secretion of pro-inflammatory cytokines, thereby resulting in intestinal inflammatory diseases [17]. Thus, the cGAS-STING-ferroptosis pathway is considered an important pathway for treating intestinal inflammatory diseases. Previous studies have shown that polyphenolic compounds such as pentagalloylglucose and punicalin reduced the expression of inflammatory cytokines in bone marrow-derived macrophages [18] and in the brains of aging mice [19] by suppressing the cGAS-STING pathway. Lv et al. [20] also found that the polyphenolic compound chlorogenic acid (CGA) improved intestinal damage of broilers challenged with NE through reducing mtDNA release and inhibiting key protein expression in the cGAS-STING pathway. Therefore, we hypothesized that CT, as a polyphenolic compound, may have the same effect as other polyphenolic compounds (such as CGA) and designed this study to investigate the effects of CT supplementation on the growth performance, intestinal morphology, intestinal barrier function, and immune function of broilers challenged with NE through the cGAS-STING-ferroptosis pathway.

## 2. Materials and Methods

### 2.1. Animal Ethical Approval

The animal experimental protocol in this study was approved by the Animal Research Ethics Committee of Qingdao Agricultural University (DKY20240527).

### 2.2. Pathogenic Bacteria, Coccidia, and CT

*C. perfringens* CVCC 2030 containing the NetB gene was supplied by the Chinese Institute for the Control of Veterinary Drugs [20]. *C. perfringens* CVCC 2030 was cultured in 50 mL of cooked meat medium at 37 °C for 24 h, and the viable count was determined to be 1 × 10^9^ CFU/mL [21]. The *Eimeria tenella*, *Eimeria necrotrix*, *Eimeria acervulina*, and *Eimeria maxima* were provided by the Qingdao Agricultural University Laboratory of Veterinary Parasitology. The CT used in this experiment is a hydrolysable tannin extracted from chestnut trees, with the content of hydrolysable tannins being 76.5% of the dry matter, purchased from Silvateam S.P.A. (San Michele di Mondovì, Italy).

### 2.3. Experimental Design and Broiler Management

In a randomized complete block design, a total of 240 one-day-old male Cobb 500 broilers (44.54 ± 0.51 g) were assigned to four groups. Each group consisted of six replicate cages (115 cm in length × 95 cm in width × 65 cm in height), with ten broilers per replicate. The four groups included a basic diet group (Control), NE challenge group (NE), 500 mg/kg CT group (L-CT), and 1000 mg/kg CT group (H-CT). On day 14, the broilers in the NE, L-CT, and H-CT groups were orally administered 1 mL saline containing 1 × 10^5^ sporulated oocysts of *Eimeria tenella*, *Eimeria necatrix*, *Eimeria acervulina*, and *Eimeria maxima*, respectively. From days 19 to 21, the above-mentioned broilers were orally administered 1 mL of bacterial solution containing 4 × 10^8^ CFU/mL of *C. perfringens* daily. The Control group received physiological saline in an equal amount. The study lasted 35 days. The basic diet met the minimum requirements according to the Cobb 500 broiler management guidelines, as shown in Table 1 [22]. No medicine was added throughout this study. All broilers were vaccinated against Newcastle Disease virus (NDV) on days 7 and 21, and for infectious bursal disease virus (IBDV) on day 14. Both feed and water were freely accessible. In the first week, the temperature was maintained at 33 to 35 °C, then decreased each week with the reduction gradually increasing, eventually dropping to approximately 23 °C. Humidity was kept at an average of 65% during the first week and 60% to 50% thereafter. At days 1 to 3, a 23 h light duration cycle was adopted, after which the light duration was adjusted to 20–22 h. The changes in the weight and feed intake of broilers were recorded during the experiment. The dead broilers were recorded and weighed daily. On days 0, 14 and 35, broilers were fasted for 12 h per cage before weighing, whereafter average daily gain (ADG), average daily feed intake (ADFI) and the feed/gain ratio (F/G) of each cage were calculated.

### 2.4. Sample Collections

On days 28 and 35, one broiler with a body weight close to the average weight of the group from each cage was selected. After fasting for 12 h, blood samples were collected from each broiler, then the serum was stored at −20 °C [23]. The duodenum, jejunum, and ileum were separated, washed with sterilized normal saline, and then fixed with 4% paraformaldehyde for histomorphometric analysis [24]. Subsequently, the remaining jejunal and ileal segments were cut longitudinally, and the intestinal mucosa was scraped for intestinal barrier and immune function analysis [23].

### 2.5. Evaluation of Fecal Coccidia Oocysts and Intestinal Lesion

On day 19, fecal samples of each cage were collected, and then the numbers of coccidia oocysts per gram (OPG) of excreta were determined [25]. Briefly, two grams of the sample were taken into a beaker and mixed with 60 mL of saturated saline solution. The mixture was subsequently filtered through a 60-mesh sieve. Then, an improved McMaster counting plate was used to count the OPG in the fecal sample. Each sample had three replicates. On days 28 and 35, duodenal, jejunal, and ileal scores were recorded [26]. All coccidia oocysts and intestinal lesion scores were determined by a single trained investigator who was blinded to the experimental group allocation.

### 2.6. Intestinal Morphological Measurements

The fixed jejunal and ileal segments were dehydrated, cleared, and embedded in paraffin to prepare sections, which were then stained with hematoxylin and eosin (HE). Then, the sections were imaged using a Zeiss Axio Scope A1 (CarlZeiss, Oberkochen, Germany) optical microscope. The Zeiss Axio Vision 4.7 image analysis software was used to measure intestinal morphology, including villus height (VH) and crypt depth (CD). Three replicates were analyzed for each sample.

### 2.7. Analysis of Biochemical Indexes

Following the steps and instructions in the manual, ELISA kits (Cusabio Co., Ltd., Wuhan, China) were used to measure the serum contents of diamine oxidase (DAO) and D-lactate (D-LA), as well as the mucosa concentrations of type I interferons (IFN-I), interferon-γ (IFN-γ), malondialdehyde (MDA), tumor necrosis factor-α (TNF-α), interleukin (IL)-10, IL-6, and IL-1β. In short, after equilibration at room temperature for 20 min, the reagents and samples were added to the wells of the plate in the prescribed volumes, then incubated in a 37 °C constant temperature chamber for 60 min. Following five washes, the chromogenic substrate was added and incubated at 37 °C in the dark for 15 min. Then, the stop solution was added to terminate the reaction. Finally, the optical density value was measured at 450 nm. A protein assay kit (Boxbio Co., Ltd., Beijing, China) was used to measure total protein content. The concentrations of D-LA, DAO, MDA, and inflammatory cytokines were standardized based on total protein concentration, and the data were expressed as the content in the mucosal protein per milligram of mucosal protein.

### 2.8. Mitochondrial Indexes Analysis

Following the method described by Lv et al. [20], genomic DNA from jejunal mucosa was extracted using the animal tissues/cells genomic DNA extraction kit (Solarbio Science & Technology Co., Ltd., Beijing, China). Subsequently, its purity and concentration were assessed by measuring the absorbance at 260/280 nm. The primer sequences (Table 2) for mitochondrially encoded NADH dehydrogenase 1 (*MT-ND1*), mitochondrially encoded NADH dehydrogenase 2 (*MT-ND2*), and mitochondrially encoded ATP synthase 6 (*MT-ATP6*) were designed using GenBank and synthesized by Genecefe Biotechnology Co., Ltd. (Wuxi, China). The reaction system was prepared according to the instructions of the qPCR Kit (Thermo Fisher Scientific Co., Ltd., Shanghai, China). Real-time fluorescence quantitative PCR detection was performed using a CFX96 real-time PCR instrument, with the following cycling conditions: 95 °C for 60 s, followed by 40 cycles of 95 °C for 5 s and 60 °C for 30 s. Three replicates were analyzed for each sample. The relative mRNA expression levels were calculated using the 2^−ΔΔCt^ method [27].

A mitochondrial extraction kit (Solarbio Science & Technology Co., Ltd., Beijing, China) and the protein assay kit (Boxbio Co., Ltd., Beijing, China) were used to isolate mitochondria from the jejunal mucosa and determine the protein concentration. The mitochondrial reactive oxygen species (mtROS) content was quantified using an ELISA kit (Keshun Science and Technology Co., Ltd., Shanghai, China), and the relative level of mtROS was normalized for further analysis.

### 2.9. Fe^2+^ Analysis

Following the method described by Zhao et al. [28], a ferrous iron colorimetric assay kit (Saint-Bio Biotechnology Co., Ltd., Shanghai, China) was used to determine Fe^2+^ concentration in the jejunal mucosal tissue. In brief, the jejunal mucosa sample was homogenized with the extraction solution and then centrifuged to obtain the supernatant for later use. The chromogenic solution was mixed with the sample supernatant and incubated. Following re-centrifugation, the resulting supernatants were transferred to the wells of a plate, and their absorbance was measured at 593 nm. A standard curve was used to calculate the Fe^2+^ concentration.

### 2.10. Western Blot Analysis

Total protein was extracted from the jejunal mucosa using RIPA lysis buffer. After SDS-PAGE, protein lysates were transferred onto a PVDF membrane and sealed with protein-free rapid blocking buffer for 10 min. After washing, the primary antibodies were added and incubated overnight at 4 °C. The membrane was washed again and incubated for 1 h with HRP-labeled anti-rabbit IgG (Real-Times Biotechnology Co., Ltd., Beijing, China). These primary antibodies targeted β-actin (AF7018), occludin (AF4605), zonula occludens-1 (ZO-1, AF5145), cGAS (DF12574), STING (DF12090), phospho-TANK binding kinase 1 (p-TBK1, AF8190), TBK1 (DF7026), phospho-interferon regulatory factor 7 (p-IRF7, AF8486), IRF7 (DF7503), phospho-nuclear factor kappa B (p-NF-kB, AF2006), NF-kB (AF0874), WD repeat domain phosphoinositide-interacting protein 2 (WIPI2, AF2734), nuclear receptor co activator factor 4 (NCOA4, DF4255), acyl-CoA synthetase long-chain family member 4 (ACSL4, DF12141), glutathione peroxidase 4 (GPX4, DF6701), ferritin heavy chain 1 (FTH1, DF6278), prostaglandin-endoperoxide synthase 2 (PTGS2) (AF7003), ferritin light chain (FTL, DF6604), ferroportin 1 (FPN1, DF13561), all purchased from Affinity Biosciences Co., Ltd. (Liyang, China); autophagy-related protein 5 (ATG5, bs-4005R) from Bioss Biotechnology Co., Ltd. (Beijing, China); and ferroptosis suppressor protein 1 (FSP1, GB115322) from Servicebio Technology Co., Ltd. (Wuhan, China). The primary antibodies were diluted with TBST buffer (G2150, Servicebio Technology Co., Ltd., Wuhan, China). The β-actin was probed on the same membrane. The protein bands were imaged using a scanner (CanoScan LiDE 100, Tokyo, Japan), and then the grayscale values of protein bands were analyzed with ImageJ (1.53k) and standardized based on β-actin.

### 2.11. Statistical Analysis

Each cage was the experimental unit for growth performance and fecal coccidia OPG counts. The broiler selected from each cage was considered as an experimental unit for analysis of intestinal lesion scores, intestinal morphology, intestinal barrier function, immune function, mitochondrial index, and the protein expression of the cGAS-STING-ferroptosis pathway. The normality of the data was analyzed by the Shapiro–Wilk test. IBM SPSS Statistics 21.0 was used for statistical analysis, employing a one-way ANOVA with Duncan’s multiple range test to evaluate the experimental results. A nonparametric test (Kruskal–Wallis) was utilized to analyze the coccidia oocyst number of excreta and intestinal lesion scores. The results were expressed as an average value, and *p* < 0.05 was defined as statistically significant.

## 3. Results

### 3.1. Fecal Coccidia Oocysts and Intestinal Lesion

As shown in Table 3, on day 19, the NE challenge led to an increase (*p* = 0.010) in OPG counts. However, compared to the NE group, the OPG in the L-CT and H-CT groups decreased (*p* < 0.001) by 53.4% and 60.1%, respectively. On days 28 and 35, the NE group showed increased (*p* < 0.001) duodenal, jejunal, and ileal lesion scores. However, on day 28, compared with the NE group, the L-CT and H-CT groups had significantly lower (*p* < 0.001) lesion scores in the duodenum (by 52.2% and 91.3%, respectively), jejunum (by 56.5% and 82.6%, respectively), and ileum (by 52.4% and 85.7%, respectively). On day 35, compared to the NE group, the L-CT and H-CT groups showed reduced (*p* < 0.001) lesion scores in the duodenum (by 56.5% and 91.3%, respectively), jejunum (by 69.2% and 96.2%, respectively), and ileum (by 64.0% and 88.0%, respectively). The lesion scores of the duodenum, jejunum, and ileum in the H-CT group decreased (*p* < 0.001) compared to those in the L-CT group on days 28 (81.8%, 60.0%, and 70.0%, respectively) and 35 (80.0%, 87.5%, and 66.7%, respectively).

### 3.2. Growth Performance

Table 4 demonstrated that there were no statistically notable variations (*p* > 0.05) in growth performance of the groups from days 0 to 14. In the NE group, compared with the Control group, the ADG was decreased by 24.4% and 19.1%, respectively, and the F/G ratio was increased (*p* < 0.001) by 11.2% and 7.1% from days 14 to 35 (*p* = 0.021) and 0 to 35 (*p* = 0.020), respectively. The ADG in the L-CT and H-CT groups increased (*p* = 0.021; 22.1% and 26.2%, respectively), and the F/G ratio was decreased (*p* < 0.001; 5.1% and 7.4%, respectively) compared to those in the NE group from days 14 to 35. The L-CT and H-CT groups had higher ADG (*p* = 0.020; increased by 16.4% and 19.2%, respectively) and lower F/G ratio (*p* < 0.001; decreased by 3.4% and 5.4%, respectively) compared with the NE group from days 0 to 35.

### 3.3. Intestinal Morphology and Barrier Function

**Figure 1:**
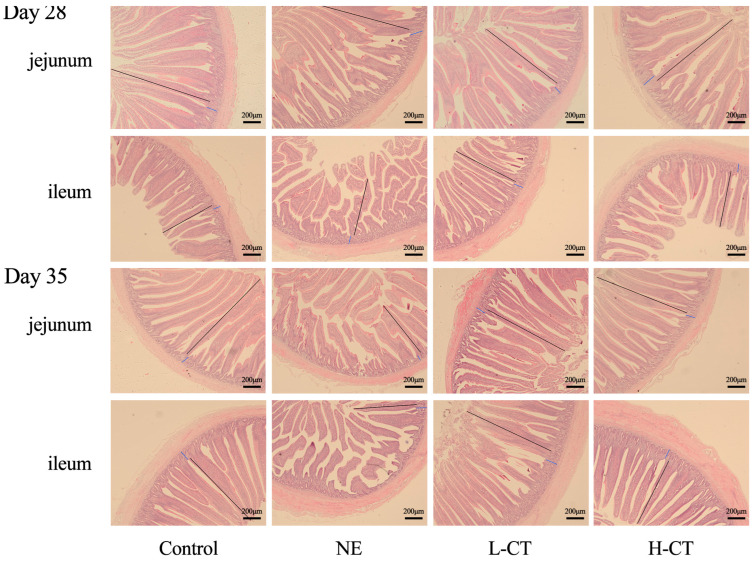
Effect of chestnut tannin (CT) on the intestinal morphology of broilers challenged with necrotic enteritis (NE) (*n* = 6 replicates per treatment), scale bar: 200 μm. Control, basic diet; NE, basic diet + NE challenge; L-CT, basic diet + NE challenge + 500 mg/kg CT; H-CT, basic diet + NE challenge + 1000 mg/kg CT. Images of the transverse section were photographed under 50× magnification.

The NE challenge altered the intestinal morphology of broilers (Figure 1). Specifically, on day 28, compared with the Control group, the jejunal CD (*p* = 0.049) and ileal CD (*p* = 0.033) in the NE group increased by 11.3% and 18.6%, respectively, while the jejunal VH (*p* = 0.001), jejunal villus height/crypt depth (V/C) ratio (*p* = 0.001), ileal VH (*p* = 0.013), and V/C ratio (*p* = 0.004) decreased by 19.3%, 27.5%, 12.6%, and 26.1%, respectively. However, compared to the NE group, the L-CT and H-CT groups increased the jejunal VH (*p* = 0.001), jejunal V/C ratio (*p* = 0.001), and ileal V/C ratio (*p* = 0.004) by 11.6%, 16.5%, 15.9%, 25.4%, 16.5%, and 26.1%, respectively. Additionally, the ileal VH in the H-CT group increased (*p* = 0.013) by 10.2%, and the jejunal CD (*p* = 0.049) and ileal CD (*p* = 0.033) decreased by 7.2% and 12.6%, respectively. On day 35, the NE group increased jejunal CD (*p* = 0.004) and ileal CD (*p* = 0.002) by 9.5% and 23.4% and decreased (*p* < 0.001) jejunal VH, ileal VH, jejunal V/C, and ileal V/C by 19.5%, 10.8%, 26.4%, and 27.1%, respectively. The jejunal VH increased by 17.0% and 20.9%, the jejunal V/C ratio by 24.2% and 31.6%, ileal VH by 7.4% and 9.4%, and ileal V/C ratio by 27.2% and 31.3% in the L-CT and H-CT groups compared with the NE group, respectively, while the jejunal CD (*p* = 0.004) decreased by 6.0% and 8.2% and ileal CD (*p* = 0.002) by 15.8% and 17.1%, respectively (Table 5).

**Figure 2:**
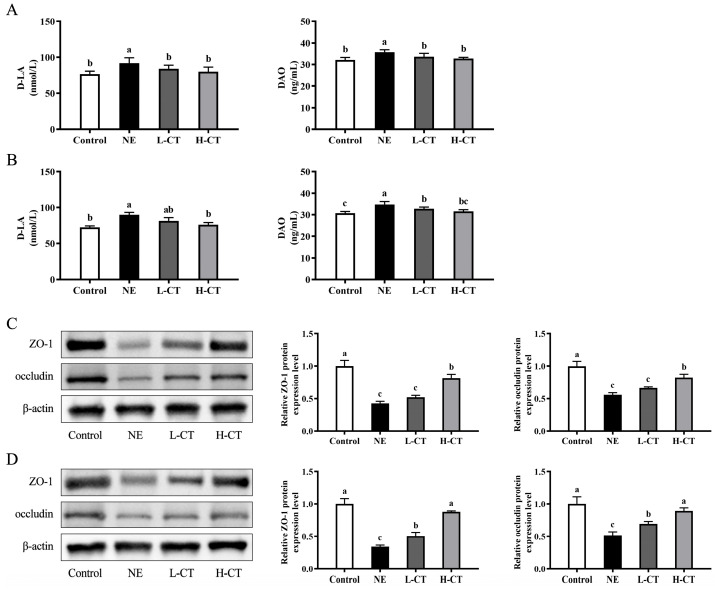
Effect of chestnut tannin (CT) on the intestinal barrier function of broilers challenged with necrotic enteritis (NE) (*n* = 6 replicates per treatment). (**A**,**B**) Serum D-LA and DAO concentrations on days 28 and 35. (**C**,**D**) The protein expression for jejunal ZO-1 and occludin on days 28 and 35. Control, basic diet; NE, basic diet + NE challenge; L-CT, basic diet + NE challenge + 500 mg/kg CT; H-CT, basic diet + NE challenge + 1000 mg/kg CT. a,b,c Different letters represent statistically notable variations (*p* < 0.05). The original Western Blots please see Figure S1.

As shown in Figure 2, on days 28 and 35, an increase in DAO concentration (*p* = 0.001) of 11.1% and 13.2% and D-LA concentration (*p* = 0.006) of 19.7% and 24.1% in the serum was observed in the NE group, respectively. Compared with the NE group, the serum D-LA concentration in the L-CT group decreased (*p* = 0.006) by 8.5% on day 28, while the serum DAO concentration decreased (*p* = 0.001) by 5.8% on days 28 and 35. On days 28 and 35, in the H-CT group, the serum D-LA concentration decreased (*p* = 0.006) by 12.9% and 15.4%, and DAO concentrations decreased by 8.1% and 9.3%, respectively. On day 28, the NE, L-CT, and H-CT groups downregulated occludin (*p* = 0.001) expression by 44.3%, 33.5%, and 17.3% and ZO-1 (*p* < 0.001) expression by 57.1%, 47.9%, and 18.3% compared to the Control group, respectively. However, the H-CT group increased occludin (*p* = 0.001) and ZO-1 (*p* < 0.001) expression by 48.3% and 90.4% compared with the NE group, respectively. Additionally, on day 35, the Control, L-CT, and H-CT groups also upregulated occludin expression (*p* = 0.001) by 94.5%, 33.8%, and 72.2% and ZO-1 expression (*p* < 0.001) by 191.3%, 46.4%, and 155.2% compared with the NE group, respectively. No statistically notable variations (*p* > 0.05) between the Control and H-CT groups.

### 3.4. Immune-Related Indexes

**Figure 3:**
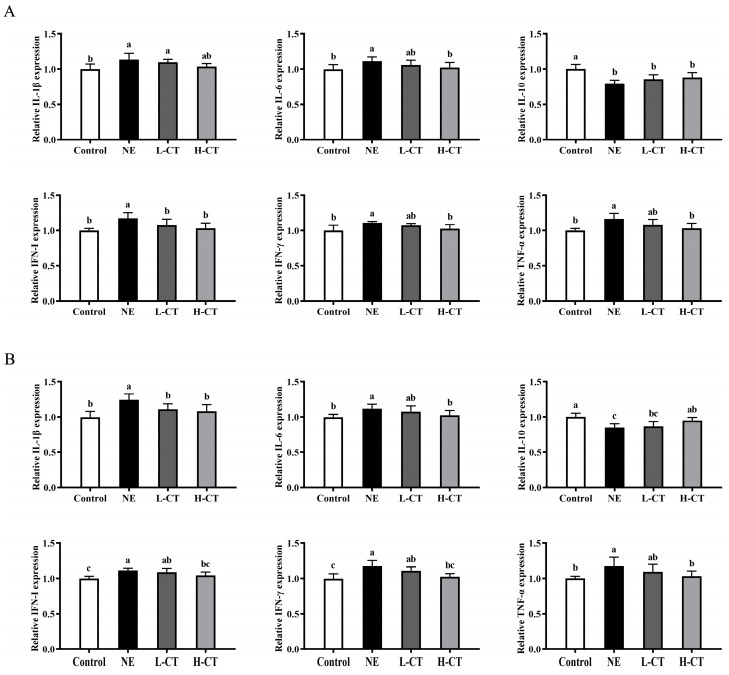
Effect of chestnut tannin (CT) on the concentrations of inflammation cytokines in jejunal mucosa of broilers challenged with necrotic enteritis (NE) on (**A**) day 28 and (**B**) day 35. (*n* = 6 replicates per treatment). Control, basic diet; NE, basic diet + NE challenge; L-CT, basic diet + NE challenge + 500 mg/kg CT; H-CT, basic diet + NE challenge + 1000 mg/kg CT. a,b,c Different letters represent statistically notable variations (*p* < 0.05).

On day 28, compared with the Control group, the levels of TNF-α (*p* = 0.013), IFN-γ (*p* = 0.044), IFN-I (*p* = 0.014), IL-6 (*p* = 0.038), and IL-1β (*p* = 0.031) increased by 16.3%, 11.1%, 17.0%, 11.2%, and 13.4% in the NE group, respectively, whereas the IL-10 was decreased (*p* = 0.013) by 20.4%. The H-CT group decreased the levels of TNF-α (*p* = 0.013), IFN-γ (*p* = 0.044), IFN-1 (*p* = 0.014), and IL-6 (*p* = 0.038) by 11.5%, 7.6%, 12.0%, and 8.0% compared to the NE group, respectively. On day 35, the levels of TNF-α (*p* = 0.039) by 14.9% and 12.2%, IFN-γ (*p* = 0.004) by 14.8% and 12.8%, IFN-I (*p* = 0.004) by 10.2% and 6.1%, IL-6 (*p* = 0.039) by 10.5% and 8.5%, and IL-1β (*p* = 0.001) by 19.6% and 12.9% in the Control and H-CT groups were reduced compared to the NE group, respectively. Additionally, the Control and H-CT groups increased (*p* = 0.012) the level of IL-10 by 17.9% and 12.1%, respectively. On days 28 and 35, no statistically notable variations (*p* > 0.05) were observed in the TNF-α, IFN-γ, IFN-I, IL-6, IL-1β, and IL-10 levels between the L-CT and H-CT groups (Table 6; Figure 3).

### 3.5. Mitochondrial Indexes

**Figure 4:**
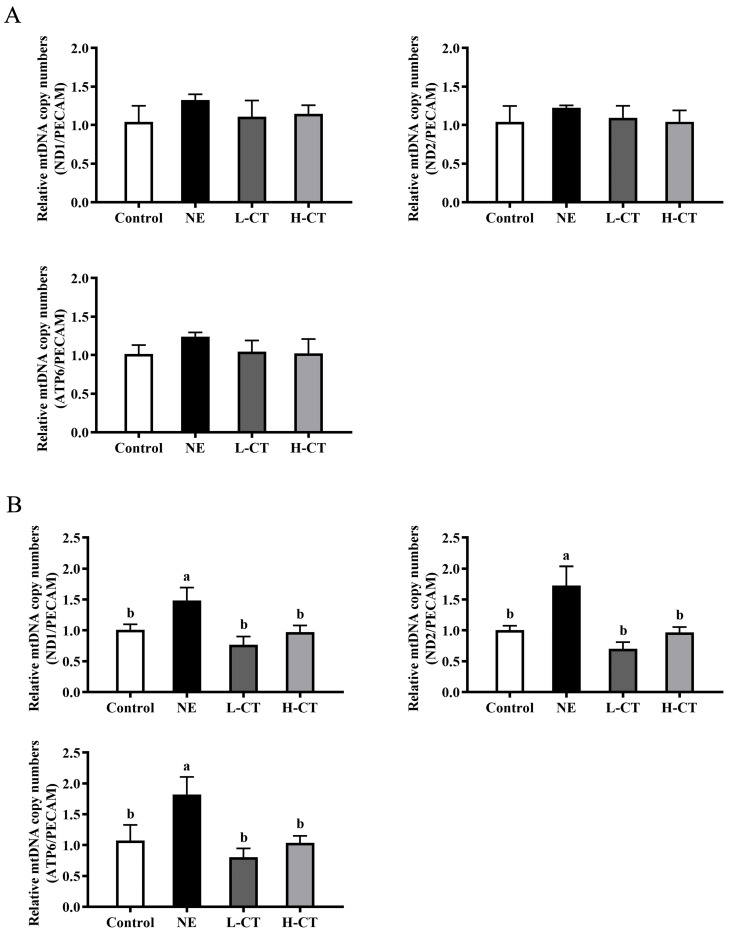
Effect of chestnut tannin (CT) on the mtDNA level in jejunal mucosa of broilers challenged with necrotic enteritis (NE) on (**A**) day 28 and (**B**) day 35. (*n* = 6 replicates per treatment). Control, basic diet; NE, basic diet + NE challenge; L-CT, basic diet + NE challenge + 500 mg/kg CT; H-CT, basic diet + NE challenge + 1000 mg/kg CT. a,b Different letters represent statistically notable variations (*p* < 0.05).

**Figure 5:**
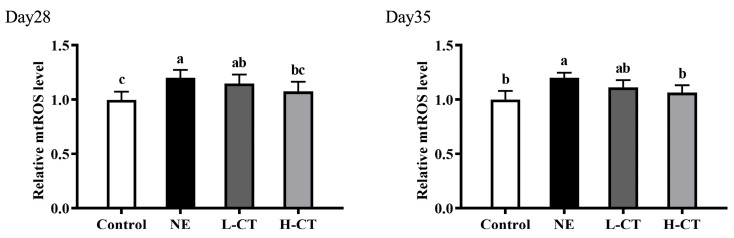
Effect of chestnut tannin (CT) on the mtROS level in the jejunal mucosa of broilers challenged with necrotic enteritis (NE) on day 28 and day 35. (*n* = 6 replicates per treatment). Control, basic diet; NE, basic diet + NE challenge; L-CT, basic diet + NE challenge + 500 mg/kg CT; H-CT, basic diet + NE challenge + 1000 mg/kg CT. a,b,c Different letters represent statistically notable variations (*p* < 0.05).

As shown in Figure 4 and Figure 5, the mtROS level on day 28 (*p* = 0.023) and 35 (*p* = 0.027) by 20.3% and 19.7% and the mRNA level of *MT-ND1* (*p* = 0.034), *MT-ND2* (*p* = 0.016), and *MT-ATP6* (*p* = 0.044) on day 35 by 47.4%, 71.7%, and 69.9% in the NE group were significantly higher compared with the Control group, respectively. Compared to the NE group, on days 28 (*p* = 0.023) and 35 (*p* = 0.027), the H-CT group decreased the level of mtROS by 10.4% and 11.0%, respectively. On day 35, the mRNA level of *MT-ND1* (*p* = 0.034) decreased by 48.5% and 34.6%, *MT-ND2* (*p* = 0.016) by 59.5% and 43.9%, and *MT-ATP6* (*p* = 0.044) by 55.9% and 43.1% in the L-CT and H-CT groups, respectively. There were no statistically notable variations (*p* > 0.05) in the mtDNA and mtROS levels in the groups outside the NE group, besides the mtROS level on day 28.

### 3.6. cGAS-STING Signaling Pathway

**Figure 6:**
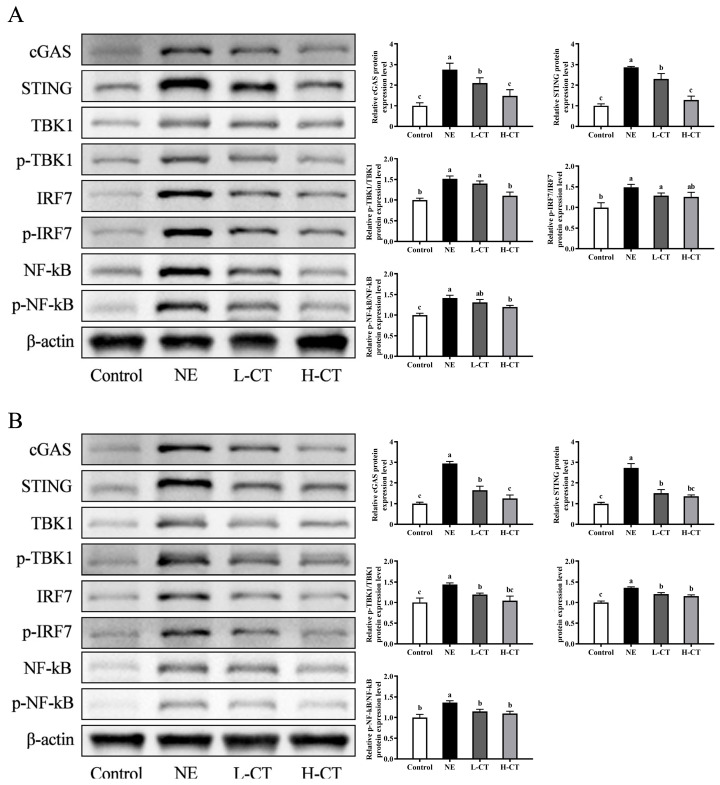
Effect of chestnut tannin (CT) on the cGAS-STING signaling pathway key protein expression in jejunal mucosa of broilers challenged with necrotic enteritis (NE) on (**A**) day 28 and (**B**) day 35. (*n* = 6 replicates per treatment). Control, basic diet; NE, basic diet + NE challenge; L-CT, basic diet + NE challenge + 500 mg/kg CT; H-CT, basic diet + NE challenge + 1000 mg/kg CT. a,b,c Different letters represent statistically notable variations (*p* < 0.05). The original Western Blots please see Figure S2.

The effects of dietary supplementation with CT on the pathway key protein expression levels are shown in Figure 6. In the NE group, on day 28, the protein expression of cGAS (*p* < 0.001), STING (*p* < 0.001), p-NF-kB/NF-kB (*p* = 0.003), p-IRF7/IRF7 (*p* = 0.017), and p-TBK1/TBK1 (*p* < 0.001) increased by 175.8%, 186.1%, 41.9%, 49.0%, and 51.8%, respectively. The NE group significantly upregulated the expression of cGAS (*p* < 0.001), STING (*p* < 0.001), p-NF-kB/NF-kB (*p* = 0.004), p-IRF7/IRF7 (*p* < 0.001), and p-TBK1/TBK1 (*p* = 0.001) by 195.0%, 174.6%, 36.6%, 35.7%, and 43.8% on day 35, respectively. On day 28, compared with the NE group, cGAS expression was downregulated by 23.6% and 46.5%, and STING expression was downregulated by 19.8% and 55.3% (*p* < 0.001) in the L-CT and H-CT groups, respectively. Additionally, the H-CT group decreased the protein expression of p-TBK1/TBK1 (*p* < 0.001) and p-NF-kB/NF-kB (*p* = 0.003) by 27.3% and 15.6%, respectively. On day 35, compared to the NE group, the expression of cGAS (*p* < 0.001) by 44.0% and 57.6%, STING (*p* < 0.001) by 44.7% and 50.6%, p-NF-kB/NF-kB (*p* = 0.004) by 15.8% and 19.4%, p-IRF7/IRF7 (*p* < 0.001) by 11.1% and 14.4%, and p-TBK1/TBK1 (*p* = 0.001) by 16.9% and 27.2% decreased in the L-CT and H-CT groups, respectively.

### 3.7. Ferroptosis-Related Indexes

On day 28, the concentration of Fe^2+^ in the Control and H-CT groups decreased (*p* = 0.030) more than that in the NE group (31.6% and 26.0%, respectively). On day 35, compared to the NE group, the Control, L-CT, and H-CT groups reduced (*p* = 0.048) Fe^2+^ concentration by 29.7%, 20.8%, and 25.7%, respectively. On days 28 (*p* = 0.045) and 35 (*p* = 0.014), the concentration of MDA was higher by 19.9% and 18.3% in the NE group compared to the Control group, respectively. The H-CT group lowered the concentration of MDA by 14.3% and 10.6% compared to the NE group on days 28 (*p* = 0.045) and 35 (*p* = 0.014), respectively. No notable variations (*p* > 0.05) in the Fe^2+^ and MDA concentrations between the Control, L-CT, and H-CT groups (Table 7).

**Figure 7:**
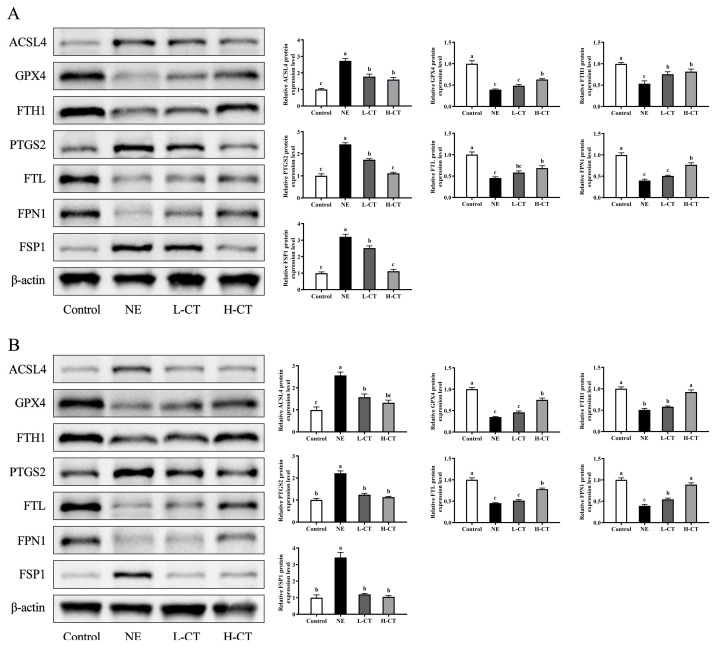
Effect of chestnut tannin (CT) on ferroptosis-related protein expression in jejunal mucosa of broilers challenged with necrotic enteritis (NE) on (**A**) day 28 and (**B**) day 35. (*n* = 6 replicates per treatment). Control, basic diet; NE, basic diet + NE challenge; L-CT, basic diet + NE challenge + 500 mg/kg CT; H-CT, basic diet + NE challenge + 1000 mg/kg CT. a,b,c Different letters represent statistically notable variations (*p* < 0.05). The original Western Blots please see Figure S3.

The effects of CT on ferroptosis-related protein expression levels are shown in Figure 7. The NE group significantly increased (*p* < 0.001) the expression of ACSL4, PTGS2, and FSP1 by 172.8%, 142.0%, and 221.5% and decreased the expression of GPX4 (*p* < 0.001), FTH1 (*p* = 0.002), FTL (*p* < 0.001), and FPN1 (*p* < 0.001) by 60.8%, 46.5%, 54.6%, and 60.2% compared to the Control group on day 28, respectively. The expression of ACSL4, PTGS2, and FSP1 was upregulated (*p* < 0.001) by 157.2%, 120.9%, and 241.1%, and the expression of GPX4, FTH1, FTL, and FPN1 was downregulated (*p* < 0.001) by 65.2%, 49.5%, 54.9%, and 60.7% in the NE group on day 35, respectively. Compared with the NE group, the protein expression of ACSL4 was 35.1% and 38.6%, PTGS2 by 28.6% and 43.9%, and FSP1 by 21.4% and 65.0% in the L-CT group on days 28 and 35 lower (*p* < 0.001), respectively, while FTH1 on day 28 (*p* = 0.002) was 40.9% and FTL on day 35 (*p* < 0.001) by 13.2% higher. On days 28 and 35, in addition to the above protein expression levels, the H-CT group also upregulated (*p* < 0.001) GPX4 expression by 60.9% and 116.6% and FPN1 expression by 94.1% and 125.0% compared with the NE group, respectively.

**Figure 8:**
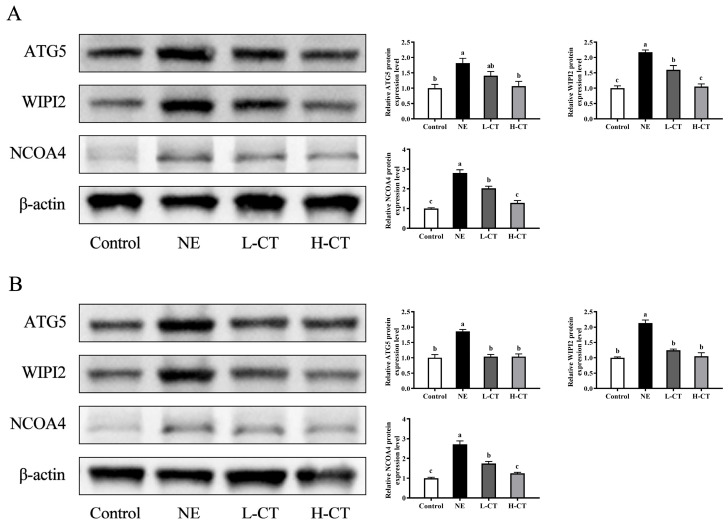
Effect of chestnut tannin (CT) on the expression of key proteins regulating ferroptosis through the cGAS-STING pathway in jejunal mucosa of broilers challenged with necrotic enteritis (NE) on (**A**) day 28 and (**B**) day 35. (*n* = 6 replicates per treatment). Control, basic diet; NE, basic diet + NE challenge; L-CT, basic diet + NE challenge + 500 mg/kg CT; H-CT, basic diet + NE challenge + 1000 mg/kg CT. a,b,c Different letters represent statistically notable variations (*p* < 0.05). The original Western Blots please see Figure S4.

Analysis of key proteins regulating ferroptosis through the above pathway revealed that NE challenge significantly upregulated ATG5 (*p* = 0.015), WIPI2 (*p* < 0.001), and NCOA4 (*p* < 0.001) expression by 82.3%, 117.5%, and 181.0% on day 28, respectively. In contrast, the L-CT and H-CT groups downregulated (*p* < 0.001) the protein expression of WIPI2 by 26.4% and 51.6% and NCOA4 by 27.5% and 54.1%, compared with the NE group, respectively. Additionally, the H-CT group upregulated (*p* < 0.05) ATG5 expression by 41.2%. On day 35, the expression of ATG5, WIPI2, and NCOA4 was upregulated (*p* < 0.001) by 85.4%, 112.9%, and 172.9% in the NE group, respectively. Compared with the NE group, the L-CT and H-CT groups downregulated (*p* < 0.001) the expression of ATG5 by 43.9%, WIPI2 by 41.5% and 50.4%, and NCOA4 by 35.9% and 54.1%, respectively (Figure 8).

## 4. Discussion

The proliferation of *C. perfringens* producing NetB is a key cause of NE, leading to cell lysis and tissue necrosis, which manifests as intestinal mucosal damage [29]. The results are consistent with Muneeb et al. [30]; this study found that *C. perfringens* and coccidia co-challenge induced severe intestinal damage, resulting in an increase in the OPG and intestinal lesion scores. Due to its relatively low molecular weight and high bioavailability, CT is more easily hydrolyzed and absorbed by the gastrointestinal tract to exert biological activity, thereby protecting the jejunal mucosa from damage [31]. In the present study, in broilers challenged with NE, dietary supplementation with CT reduced the intestinal lesion scores and OPG while improving the ADG and F/G ratio. According to a previous study, ellagic acid, a kind of hydrolysable tannin, alleviated intestinal damage and microbial imbalance in broilers challenged with *C. perfringens*, ultimately improving ADG and FCR [32], which indicates a close correlation between improvements in growth performance and the intestinal tract.

The intestinal morphology of poultry reflects the nutritional digestion and absorption function of the intestine, and its villous crypt structure is regarded as a significant indicator for evaluating normal intestinal physiological function [33]. Co-infection with *C. perfringens* and coccidia damages intestinal epithelial cells, causing villus atrophy and shedding, reducing the nutrient absorption area, thereby slowing growth [34]. Similar results were observed in the present study. However, our results indicated that dietary CT supplementation decreased the jejunal and ileal CD and increased VH and V/C ratio in broilers challenged with NE, suggesting that CT increases the contact area of intestinal villi for nutrient absorption, thereby improving the growth performance. A previous study found that CT accelerates intestinal growth in broilers by enhancing VH and V/C ratio [35]. Similarly, gallic acid, which belongs to hydrolysable tannins, significantly decreased CD and increased the V/C ratio in broiler jejunum [36]. During the process of the intestinal epithelial barrier resisting bacterial colonization and invasion, tight junction proteins and serum markers play crucial roles [37]. Unlike polyphenolic compounds such as quercetin [38] and epigallocatechin gallate [39], which indirectly regulate intestinal barrier function, CT actively acts on intestinal epithelial cells to enhance barrier integrity [40]. In this study, CT decreased the serum concentrations of D-LA and DAO of broilers challenged with NE while increasing the jejunal ZO-1 and occludin expression, suggesting that CT improves intestinal health by protecting the integrity of the jejunal barrier. These results were consistent with Li et al. [41], who found that ellagic acid supplementation increased the serum DAO content in the Escherichia coli K88-challenged broilers and upregulated the mRNA levels of *ZO-1*, *occluding*, and *claudin-1* in the jejunum and ileum.

Intestinal inflammation resulting from damage to the intestinal barrier in broilers is typically associated with cytokine regulation [42]. In the present research, CT reduced the IL-1β, IL-6, IFN-1, IFN-γ, and TNF-α concentrations in the jejunum while increasing the IL-10 concentration. Additionally, the protein expression of NF-kB was downregulated, suggesting that the effect of CT on intestinal inflammatory cytokines may be mediated through inhibition of the NF-kB signaling pathway. NF-kB transfers to the nucleus and directly binds to the promoter regions of inflammatory cytokine genes (IL-1β, IL-6, IFN-I, and IFN-γ) to induce their expression [43]. Additionally, NF-kB forms a positive feedback regulation with TNF-α to further amplify inflammatory signals [44]. A previous study pointed out that IL-1β, IL-6, and IFN-γ concentrations were increased in the jejunal mucosa of broilers challenged with NE, while IL-10 was decreased [20]. Dietary supplementation with ellagic acid downregulated the mRNA expressions of IL-1β, IL-8, and TNF-α of the jejunum in *C. perfringens*-challenged broilers through suppressing the NF-kB signaling pathway [32].

Mitochondria provide energy and metabolic support for the intestinal epithelial cells, but excessive accumulation of mtROS induces mitochondrial dysfunction, resulting in the release of mtDNA, which promotes intestinal inflammatory damage [45,46]. Studies found that mitochondrial damage in intestinal epithelial cells of broilers challenged with NE is a crucial factor that leads to downstream cell necrosis and inflammatory damage [47]. This mitochondrial damage may be caused by toxins produced by *C. perfringens* [48]. In the present study, NE challenge caused an increase in the mtROS and mtDNA levels in broilers, while the supplementation of CT reversed this effect, which indicates that CT could play a protective role in mitochondrial dysfunction. Similar studies have shown that ellagic acid and gallic acid protected mitochondrial structure and function by increasing mitochondrial membrane potential, reducing the levels of mtROS and MDA, and improving mitochondrial swelling [49,50].

The activation of the cGAS-STING pathway depends on the capacity of cGAS to recognize both exogenous and endogenous DNA within the cytoplasm [51]. The invasion of pathogens triggers mitochondrial damage in the host, inducing the release of mtDNA [52]. cGAS binds to mtDNA and induces cGAMP production, thereby activating STING [53,54]. Then, STING facilitates the recruitment of TBK1 and IKK, which promotes the phosphorylation of IRF7 and the activation of NF-kB. Subsequently, phosphorylated IRF7 and activated NF-kB translocate into the nucleus and induce the expression of IFN-I and inflammatory cytokines [55]. In the present study, NE challenge increased the protein expression of cGAS, STING, p-IRF7, and p-TBK1 in the jejunum of broilers, which were downregulated after supplementation with CT, indicating that CT effectively inhibits the cGAS-STING pathway through downregulation of key protein expression. These findings are consistent with Lv et al. [20], who suggested that CGA decreased cGAS, p-IRF7, and p-TBK1 expression levels of broilers challenged with NE, thereby inhibiting the cGAS-STING pathway. Similarly, Chen et al. [19] indicated that punicalin, an ellagitannin component, downregulated the expression of inflammatory cytokines in aging mouse brain cells via the inhibition of the cGAS-STING pathway.

Ferroptosis is a distinct mode of cell death induced by dysregulation of iron homeostasis, mitochondrial dysfunction, and lipid peroxidation, setting it apart from apoptosis, necrosis, and autophagy [56]. Autophagy of ferritin and the reduction in FPN1 lead to a large accumulation of intracellular Fe^2+^ [57], resulting in downregulation of the activity of GPX4, thereby inducing cell ferroptosis [58]. Then, damage-associated molecular patterns are released from ferroptotic cells to activate NF-kB [59]. The results in this study demonstrated that the upregulation of FTH1, FTL, and FPN1 expression reduced Fe^2+^ release, while the downregulation of Fe^2+^ concentration and FSP expression led to decreased ACSL4 expression and MDA concentration and increased GPX4 expression. Additionally, the downregulation of the PTGS2 expression directly reflected a decrease in lipid peroxidation levels. These findings indicate that CT supplementation alleviates the abnormal accumulation of Fe^2+^ and reduces lipid peroxidation by regulating the expression of ferroptosis-related proteins, thereby inhibiting ferroptosis. Previous research pointed out that ellagic acid inhibited dihydrotestosterone-induced ferroptosis in mice by reducing iron content and MDA level and increasing GPX4 expression level [60]. Ellagic acid downregulated the increase in Fe^2+^ through upregulating the protein expression of FPN1 and reduced MDA level by neutralizing ROS, thereby increasing the protein expression of GPX4 [61]. Notably, most studies targeting the cGAS-STING pathway for regulating ferroptosis suggested that this pathway provides a new avenue for the exploration of therapeutic options in the context of various diseases [62,63]. Our study demonstrated that CT supplementation alleviated the increased protein expression of ATG5, WIPI2, and NCOA4 of broilers challenged with NE, revealing that the cGAS-STING pathway is an important pathway for CT to inhibit ferroptosis. STING induces ferroptosis through an ATG5- and WIPI2-dependent pathway [64,65] and NCOA4-mediated ferritin autophagy [66]. Relies on the cGAS-STING axis; STING directly targets GPX4 to initiate autophagy-dependent ferroptosis [67], but also upregulates the expression of NCOA4 to decrease ferritin levels, thereby inducing ferroptosis [68]. Based on our results, the alleviation of intestinal inflammatory damage of broilers challenged with NE by CT is achieved via inhibiting the cGAS-STING-ferroptosis pathway.

## 5. Conclusions

Supplementing CT may improve the growth performance, intestinal morphology and barrier function, and immune function of broilers challenged with NE by attenuating the signal transmission of the cGAS-STING-ferroptosis pathway, which was more effective at a dose of 1000 mg/kg in this study. These findings provide a new intervention target in nutritional regulation of intestinal inflammatory damage in broilers challenged with NE. However, future studies are needed to confirm the direct link of the cGAS-STING-ferroptosis pathway by using specific inhibitors (such as the STING inhibitor C-176). Additionally, multiple signaling pathways are involved in NE infection, and CT may have multiple targets in the treatment. Therefore, further studies are needed on additional pathways of CT to protect broilers challenged with NE.

## Figures and Tables

**Table 1:** Composition and nutrient content of basal diets (air-dry basis) %.

Items	0–21 Days of Age	22–35 Days of Age
Ingredients		
Corn	55.70	60.32
Soybean meal	33.71	29.59
Fish meal	3.40	3.08
Soybean oil	3.54	3.65
Limestone	1.32	1.28
CaHPO_4_	0.51	0.33
NaCl	0.30	0.30
DL-Methionine	0.36	0.31
L-lysine	0.15	0.13
Premix ^1^	1.00	1.00
Total	100	100
Total nutrient levels ^2^		
Metabolizable energy (MJ/kg)	12.58	12.79
Crude protein, %	21.56	19.97
Calcium, %	0.95	0.85
Available phosphorus, %	0.69	0.50
Lysine, %	1.28	1.15
Methionine, %	0.49	0.45
Threonine, %	0.83	0.76
Tryptophan, %	0.25	0.23

^1^ Per kilogram of dietary supply: iron, 40 mg; manganese, 90 mg; zinc, 75 mg; copper, 8 mg; iodine, 0.20 mg; selenium, 0.40 mg; vitamin A (trans-retinyl acetate), 7000 IU; vitamin B_1_ (thiamin), 0.50 mg; vitamin B_2_ (riboflavin), 8 mg; vitamin B_6_ (pyridoxine HCI), 4 mg; vitamin B_12_ (cobalamin), 0.02 mg; vitamin D_3_ (cholecalciferol), 4000 IU; vitamin E (all-rac-α-tocopherol acetate), 72 IU; vitamin K_3_ (menadione), 3.5 mg; nicotinic acid, 36 mg; calcium pantothenate, 9.0 mg; folic acid, 2.50 mg; biotin, 0.10 mg. ^2^ The nutrient levels were calculated values.

**Table 2:** The primer used for real-time PCR.

Genes ^1^	Accession No.	Primer Sequence (5′ to 3′)	Product Size (bp)
*β-actin*	NC_052545.1	F: GCTACGTCGCACTGGATTTCG	125
		R: GGGGCACCTGAACCTCTCATT	
*MT-ND1*	NC_053523.1	F: ACCACCGTCCTATTCCTGAACC	133
		R: AGCGGAACCGTGGATATGAGG	
*MT-ND2*	NC_053523.1	F: AGCTGGCCTCCCACCATTAAC	146
		R: AGTGGTATGCAAGTCGGAGGT	
*MT-ATP6*	NC_053523.1	F: GGCCATTAGCCCTAGGAGTACG	87
		R: GGGCGATTGTGGCTGTAGAGA	

^1^ *MT-ND1*: mitochondrially encoded NADH dehydrogenase 1; *MT-ND2*: mitochondrially encoded NADH dehydrogenase 2; *MT-ATP6*: mitochondrially encoded Adenosine Triphosphate 6.

**Table 3:** Effect of chestnut tannin (CT) on the coccidia oocysts per gram of excreta and intestinal lesion scores of broilers challenged with necrotic enteritis (NE) ^1^.

Items ^2^	Control ^3^	NE	L-CT	H-CT	SEM ^4^	Kruskal–Wallis *H* Value	*p*-Value
Day 19							
OPG, ×10^6^/g of excreta	0.00 ^c^	1.74 ^a^	0.81 ^b^	0.70 ^b^	0.141	20.983	<0.001
Day 28							
Duodenum	0.00 ^c^	3.83 ^a^	1.83 ^b^	0.33 ^c^	0.335	20.486	<0.001
Jejunum	0.00 ^c^	3.83 ^a^	1.67 ^b^	0.67 ^c^	0.324	19.981	<0.001
Ileum	0.00 ^c^	3.50 ^a^	1.67 ^b^	0.50 ^c^	0.294	20.552	<0.001
Day 35							
Duodenum	0.00 ^c^	3.83 ^a^	1.67 ^b^	0.33 ^c^	0.340	20.328	<0.001
Jejunum	0.00 ^c^	4.33 ^a^	1.33 ^b^	0.17 ^c^	0.371	21.166	<0.001
Ileum	0.00 ^c^	4.17 ^a^	1.50 ^b^	0.50 ^c^	0.351	20.206	<0.001

^1^ *n* = 6 replicates per treatment. ^2^ OPG, oocysts per gram of excreta. ^3^ Control, control diet; NE, control diet + NE challenge; L-CT, control diet + NE challenge + 500 mg/kg CT; H-CT, control diet + NE challenge + 1000 mg/kg CT. ^4^ SEM, standard error means. ^a,b,c^ Different superscripts indicate significant differences (*p* < 0.05).

**Table 4:** Effect of chestnut tannin (CT) on the growth performance of broilers challenged with necrotic enteritis (NE) ^1^.

Items ^2^	Control ^3^	NE	L-CT	H-CT	SEM ^4^	*p*-Value
Day 0–14						
ADG (g)	35.97	35.48	36.35	36.49	0.223	0.401
ADFI (g)	40.47	39.65	40.18	39.95	0.273	0.778
F/G	1.13	1.12	1.11	1.10	0.006	0.266
Day 14–35						
ADG (g)	86.15 ^a^	65.10 ^b^	79.51 ^a^	82.16 ^a^	2.692	0.021
ADFI (g)	134.23 ^a^	112.84 ^b^	131.01 ^ab^	131.73 ^ab^	3.767	0.161
F/G	1.56 ^d^	1.74 ^a^	1.65 ^b^	1.61 ^c^	0.014	<0.001
Day 0–35						
ADG (g)	65.51 ^a^	53.01 ^b^	61.70 ^a^	63.17 ^a^	1.594	0.020
ADFI (g)	95.66 ^a^	82.97 ^b^	93.51 ^ab^	93.60 ^ab^	2.229	0.174
F/G	1.46 ^c^	1.57 ^a^	1.51 ^b^	1.48 ^bc^	0.009	<0.001

^1^ *n* = 6 replicates per treatment. ^2^ ADG, average daily gain; ADFI, average daily feed intake; F/G, feed/gain ratio. ^3^ Control, control diet; NE, control diet + NE challenge; L-CT, control diet + NE challenge + 500 mg/kg CT; H-CT, control diet + NE challenge + 1000 mg/kg CT. ^4^ SEM, standard error means. ^a,b,c,d^ Different superscripts indicate significant differences (*p* < 0.05).

**Table 5:** Effect of chestnut tannin (CT) on the intestinal morphology of broilers challenged with necrotic enteritis (NE) ^1^.

Items ^2^	Control ^3^	NE	L-CT	H-CT	SEM ^4^	*p*-Value
Day 28						
Jejunum						
VH (μm)	1208.13 ^a^	975.22 ^c^	1088.35 ^b^	1136.12 ^ab^	27.317	0.001
CD (μm)	158.99 ^b^	176.92 ^a^	170.39 ^ab^	164.27 ^b^	2.596	0.049
V/C	7.63 ^a^	5.54 ^c^	6.41 ^b^	6.94 ^ab^	0.249	0.001
Ileum						
VH (μm)	846.52 ^a^	739.78 ^c^	789.05 ^bc^	814.84 ^ab^	13.875	0.013
CD (μm)	146.07 ^b^	173.19 ^a^	158.39 ^ab^	151.31 ^b^	3.823	0.033
V/C	5.81 ^a^	4.30 ^c^	5.01 ^b^	5.42 ^ab^	0.190	0.004
Day 35						
Jejunum						
VH (μm)	1344.14 ^a^	1082.66 ^b^	1266.17 ^a^	1308.73 ^a^	32.080	<0.001
CD (μm)	168.28 ^b^	184.22 ^a^	173.21 ^b^	169.13 ^b^	2.152	0.004
V/C	8.00 ^a^	5.89 ^c^	7.32 ^b^	7.75 ^ab^	0.254	<0.001
Ileum						
VH (μm)	912.00 ^a^	813.73 ^c^	874.14 ^b^	890.31 ^ab^	11.725	<0.001
CD (μm)	145.02 ^b^	178.96 ^a^	150.65 ^b^	148.28 ^b^	4.519	0.002
V/C	6.30 ^a^	4.59 ^b^	5.84 ^a^	6.03 ^a^	0.209	<0.001

^1^ *n* = 6 replicates per treatment. ^2^ VH, villus height; CD, crypt depth; V/C, villus height/crypt depth. ^3^ Control, control diet; NE, control diet + NE challenge; L-CT, control diet + NE challenge + 500 mg/kg CT; H-CT, control diet + NE challenge + 1000 mg/kg CT. ^4^ SEM, standard error means. ^a,b,c^ Different superscripts indicate significant differences (*p* < 0.05).

**Table 6:** Effect of chestnut tannin (CT) on the immune-related indexes concentrations of broilers challenged with necrotic enteritis (NE) ^1^.

Items ^2^	Control ^3^	NE	L-CT	H-CT	SEM ^4^	*p*-Value
Day 28						
IL-1β (pg/mgprot)	38.80 ^b^	44.00 ^a^	42.65 ^a^	40.26 ^ab^	0.746	0.031
IL-6 (pg/mgprot)	13.66 ^b^	15.18 ^a^	14.47 ^ab^	13.97 ^b^	0.207	0.038
IL-10 (pg/mgprot)	16.05 ^a^	12.77 ^b^	13.74 ^b^	14.18 ^b^	0.355	0.013
IFN-I (pg/mgprot)	46.09 ^b^	53.92 ^a^	49.68 ^ab^	47.47 ^b^	0.957	0.014
IFN-γ (pg/mgprot)	76.41 ^b^	84.88 ^a^	82.07 ^ab^	78.41 ^b^	1.262	0.044
TNF-α (pg/mgprot)	16.89 ^b^	19.64 ^a^	18.23 ^ab^	17.39 ^b^	0.335	0.013
Day 35						
IL-1β (pg/mgprot)	31.76 ^b^	39.50 ^a^	35.35 ^b^	34.41 ^b^	0.827	0.001
IL-6 (pg/mgprot)	11.79 ^b^	13.18 ^a^	12.70 ^ab^	12.06 ^b^	0.194	0.039
IL-10 (pg/mgprot)	13.65 ^a^	11.58 ^c^	11.81 ^bc^	12.98 ^ab^	0.278	0.012
IFN-I (pg/mgprot)	42.10 ^c^	46.88 ^a^	45.76 ^ab^	44.02 ^bc^	0.575	0.004
IFN-γ (pg/mgprot)	70.87 ^c^	83.16 ^a^	78.78 ^ab^	72.49 ^bc^	1.590	0.004
TNF-α (pg/mgprot)	12.84 ^b^	15.09 ^a^	14.03 ^ab^	13.24 ^b^	0.312	0.039

^1^ *n* = 6 replicates per treatment. ^2^ IL, interleukin; TNF-α, tumor necrosis factor-α; IFN-γ, interferon-γ; IFN-I, Type I interferon. ^3^ Control, control diet; NE, control diet + NE challenge; L-CT, control diet + NE challenge + 500 mg/kg CT; H-CT, control diet + NE challenge + 1000 mg/kg CT. ^4^ SEM, standard error means. ^a,b,c^ Different superscripts indicate significant differences (*p* < 0.05).

**Table 7:** Effect of chestnut tannin (CT) on Fe^2+^ and MDA levels in jejunal mucosa in broilers challenged with NE ^1^.

Items ^2^	Control ^3^	NE	L-CT	H-CT	SEM ^4^	*p*-Value
Day 28						
Fe^2+^ (μmol/kgprot)	123.26 ^b^	180.16 ^a^	155.69 ^ab^	133.33 ^b^	7.641	0.030
MDA (nmol/gprot)	2.37 ^b^	2.84 ^a^	2.50 ^ab^	2.43 ^b^	0.725	0.045
Day 35						
Fe^2+^ (μmol/kgprot)	118.16 ^b^	168.08 ^a^	133.08 ^b^	124.82 ^b^	6.666	0.048
MDA (nmol/gprot)	2.08 ^b^	2.46 ^a^	2.28 ^ab^	2.19 ^b^	0.473	0.014

^1^ *n* = 6 replicates per treatment. ^2^ Fe^2+^, ferrous ions; MDA, malondialdehyde. ^3^ Control, control diet; NE, control diet + NE challenge; L-CT, control diet + NE challenge + 500 mg/kg CT; H-CT, control diet + NE challenge + 1000 mg/kg CT. ^4^ SEM, standard error means. ^a,b^ Different superscripts indicate significant differences (*p* < 0.05).

## Data Availability

All data in this study are available upon request from the corresponding author.

## Supplementary Materials

The following supporting information can be downloaded at: https://www.mdpi.com/article/10.3390/ani16040686/s1, Figure S1: Original image of Western blot in Figure 2; Figure S2: Original image of Western blot in Figure 6; Figure S3: Original image of Western blot in Figure 7; Figure S4: Original image of Western blot in Figure 8.
