# Supplementary material for: Chestnut Tannin Improves Growth Performance and Intestinal Health of Broilers Challenged with Necrotic Enteritis via the cGAS-STING-Ferroptosis Pathway
Source: Animals (Basel). 2026 Feb 22;16(4):686. doi: 10.3390/ani16040686 (PMC12937207; doi:10.3390/ani16040686)
Supplement: Supplementary file 1 [file animals-16-00686-s001.zip › animals-4150724-supplementary.pdf]

Chestnut tannin improves growth performance and intestinal health of broilers challenged with necrotic enteritis via the cGAS-STING-ferroptosis pathway

Genrui Zhang, Fandi Tang, Yang Wang, Huawei Liu *

The name of the journal: *animals* journal

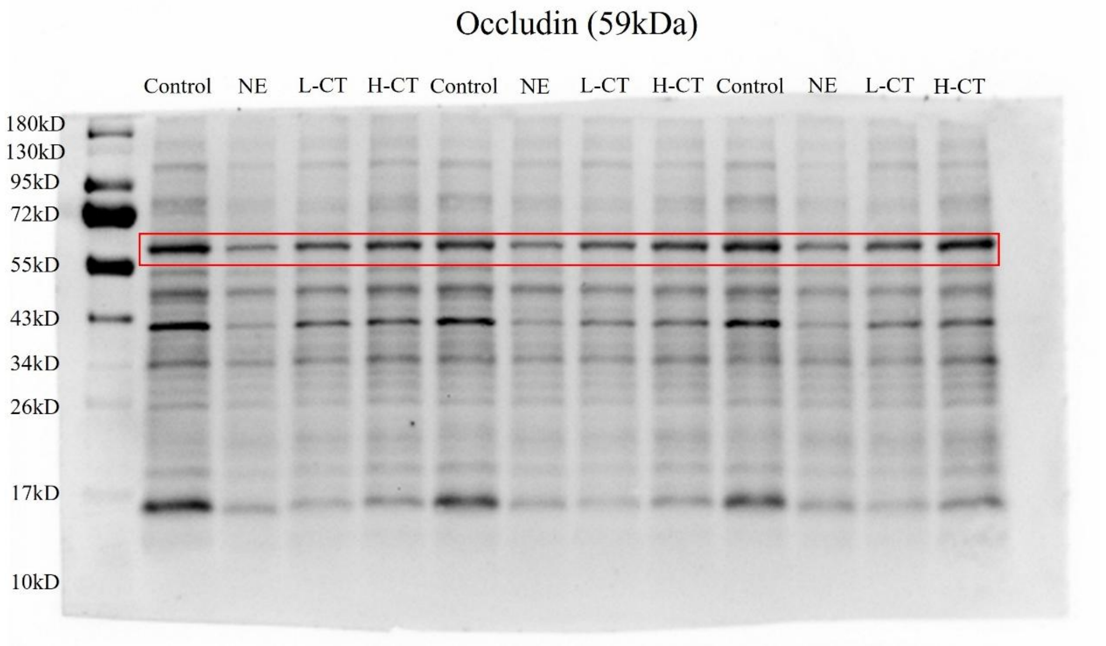

Supplementary Figure S1 (a) The full membrane with molecular weight ladder used for western blot analysis of Occludin in the jejunum of broilers challenged with necrotic enteritis for day 28 in Figure 2.

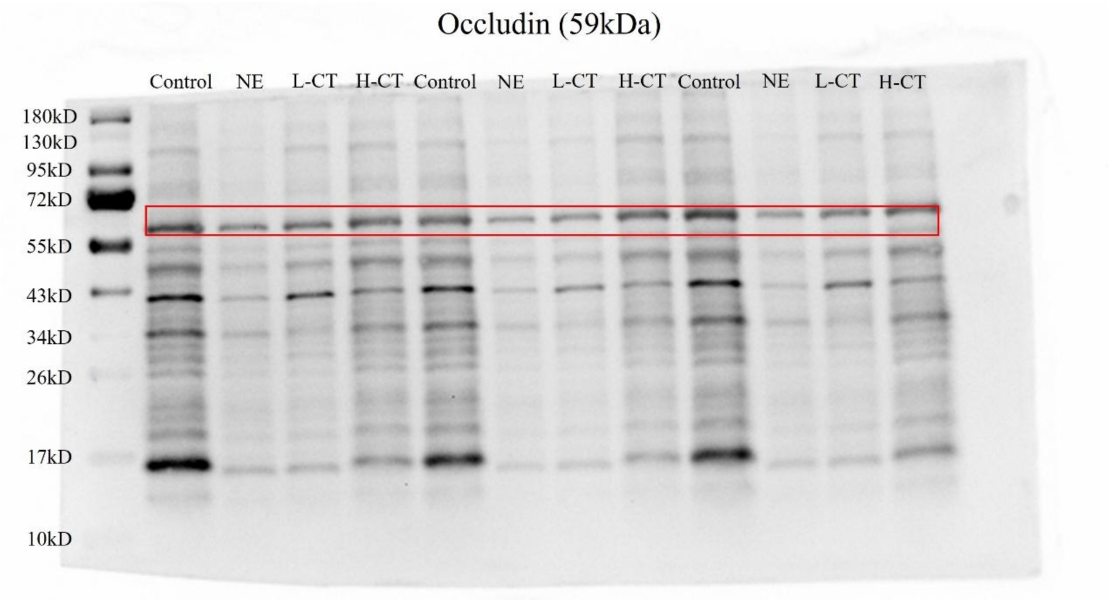

Supplementary Figure S1 (b) The full membrane with molecular weight ladder used for western blot analysis of Occludin in the jejunum of broilers challenged with necrotic enteritis for day 35 in Figure 2.

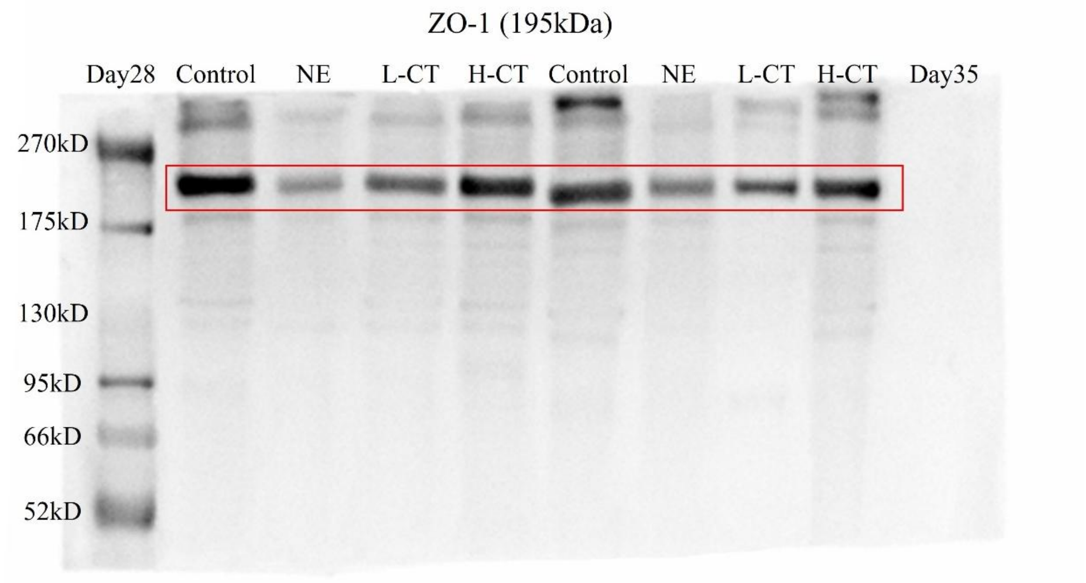

Supplementary Figure S1 (c) The full membrane with molecular weight ladder used for western blot analysis of ZO-1 in the jejunum of broilers challenged with necrotic enteritis for days 28 and 35. The protein bands from left to right are as follows: Control (day 28), NE (day 28), L-CT (day 28), H-CT (day 28), Control (day 35), NE (day 35), L-CT (day 35) and H-CT (day 35) in Figure 2.

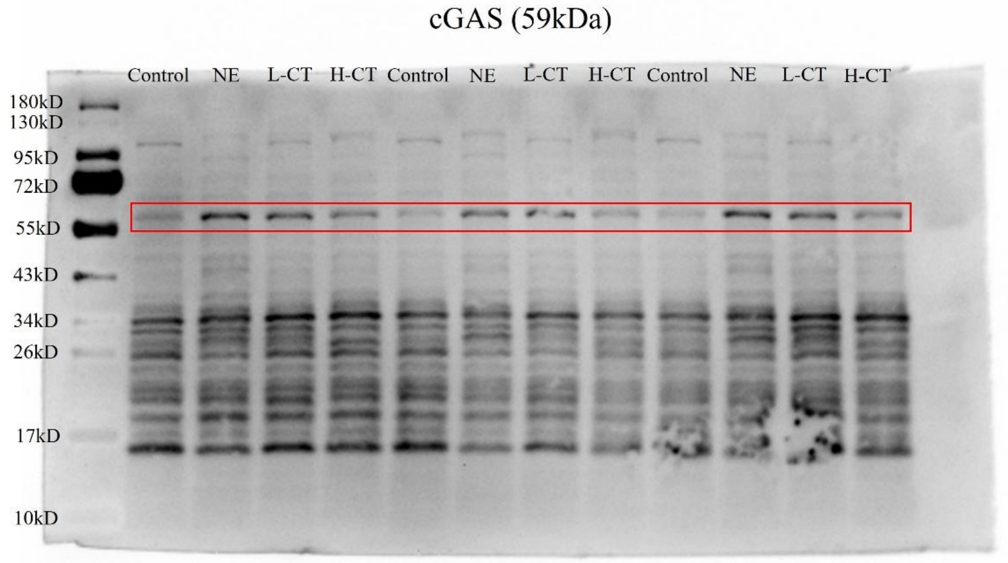

Supplementary Figure S2 (a) The full membrane with molecular weight ladder used for western blot analysis of cGAS in the jejunum of broilers challenged with necrotic enteritis for day 28 in Figure 6.

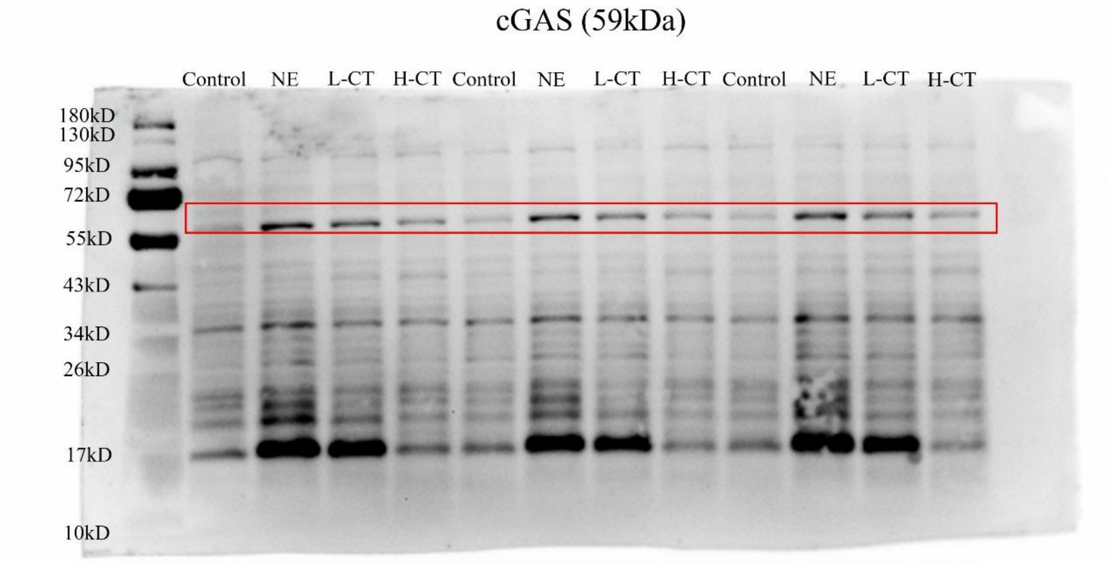

Supplementary Figure S2 (b) The full membrane with molecular weight ladder used for western blot analysis of cGAS in the jejunum of broilers challenged with necrotic enteritis for day 35 in Figure 6.

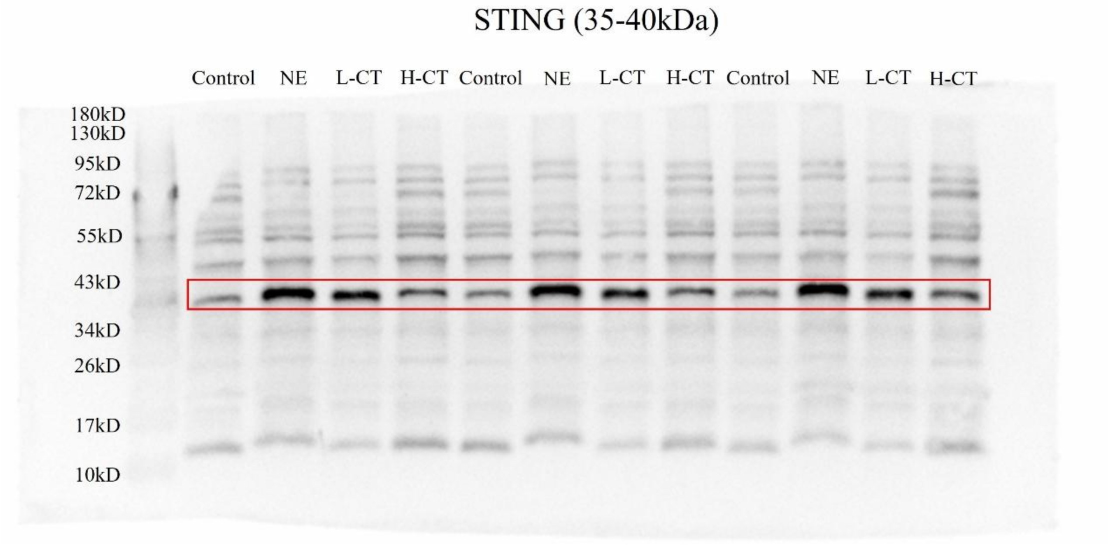

Supplementary Figure S2 (c) The full membrane with molecular weight ladder used for western blot analysis of STING in the jejunum of broilers challenged with necrotic enteritis for day 28 in Figure 6.

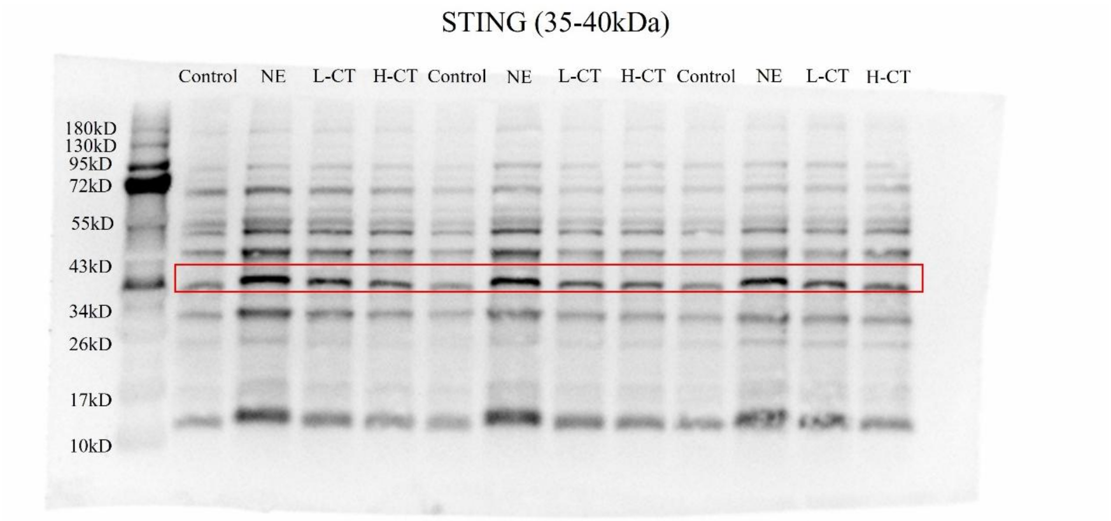

Supplementary Figure S2 (d) The full membrane with molecular weight ladder used for western blot analysis of STING in the jejunum of broilers challenged with necrotic enteritis for day 35 in Figure 6.

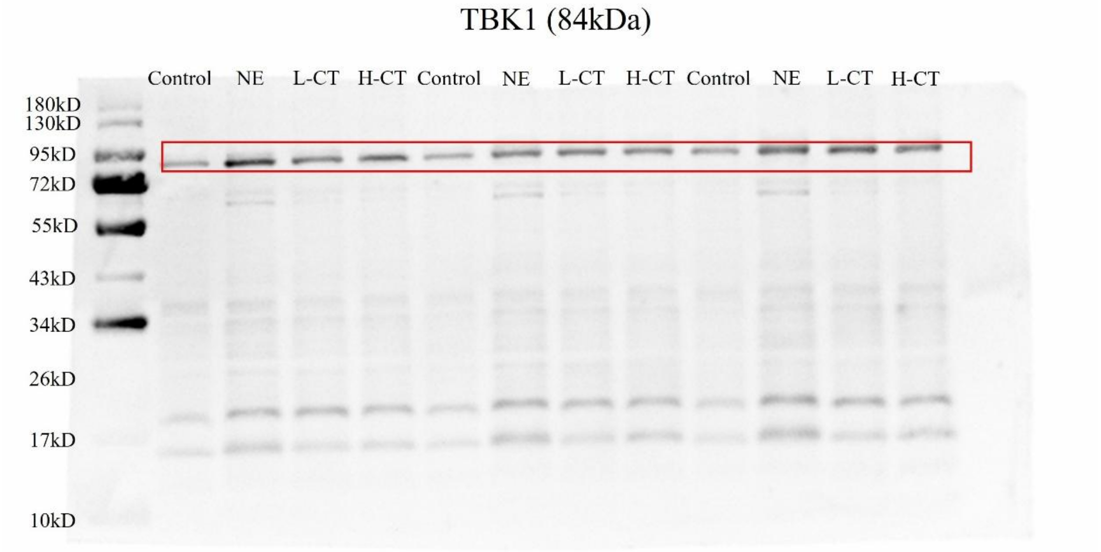

Supplementary Figure S2 (e) The full membrane with molecular weight ladder used for western blot analysis of TBK1 in the jejunum of broilers challenged with necrotic enteritis for day 28 in Figure 6.

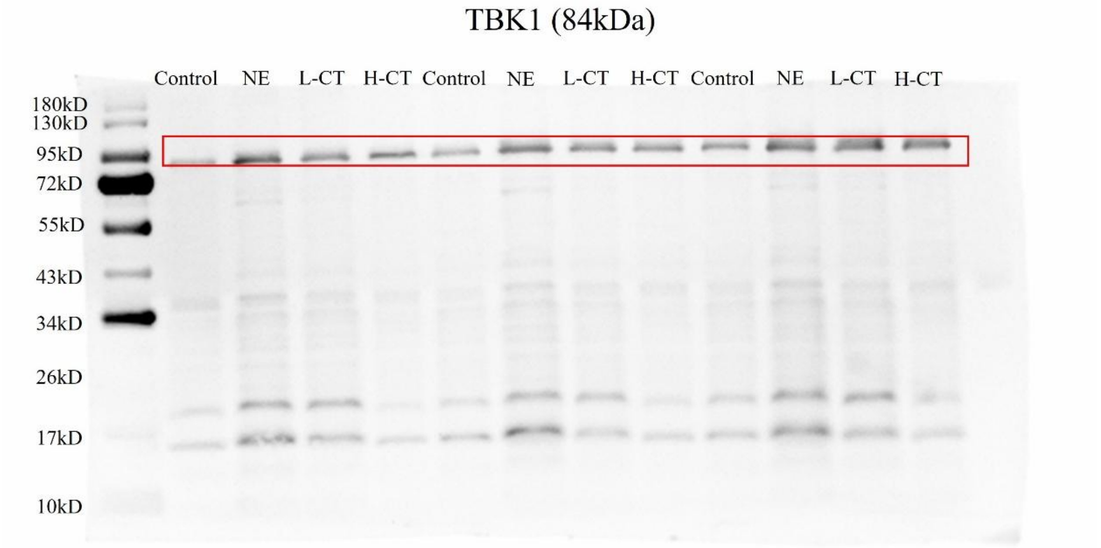

Supplementary Figure S2 (f) The full membrane with molecular weight ladder used for western blot analysis of TBK1 in the jejunum of broilers challenged with necrotic enteritis for day 35 in Figure 6.

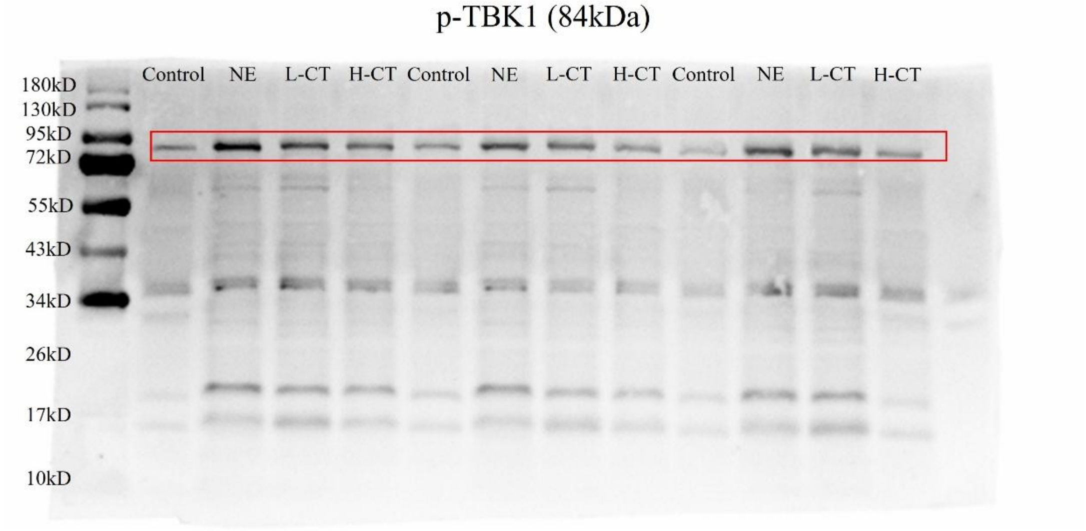

Supplementary Figure S2 (g) The full membrane with molecular weight ladder used for western blot analysis of p-TBK1 in the jejunum of broilers challenged with necrotic enteritis for day 28 in Figure 6.

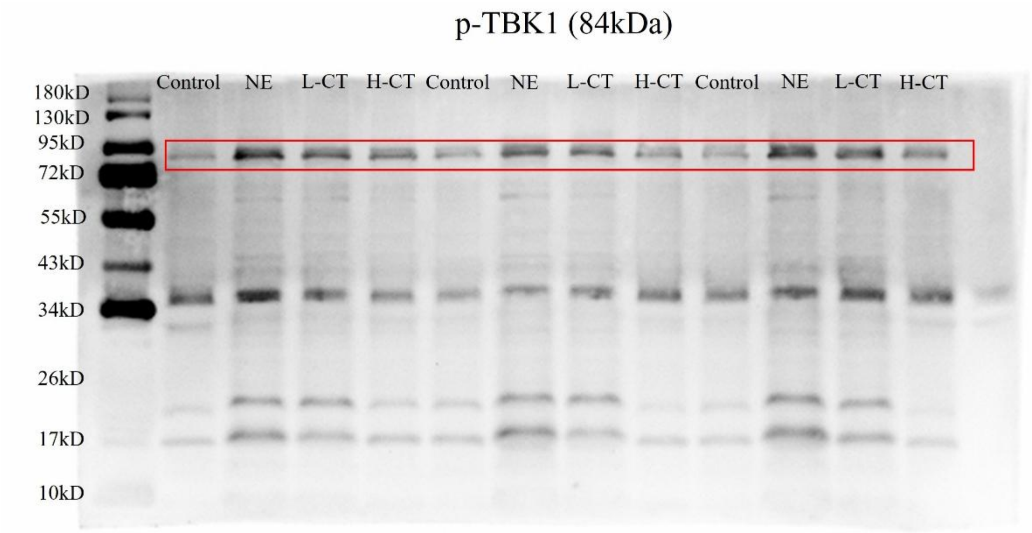

Supplementary Figure S2 (h) The full membrane with molecular weight ladder used for western blot analysis of p-TBK1 in the jejunum of broilers challenged with necrotic enteritis for day 35 in Figure 6.

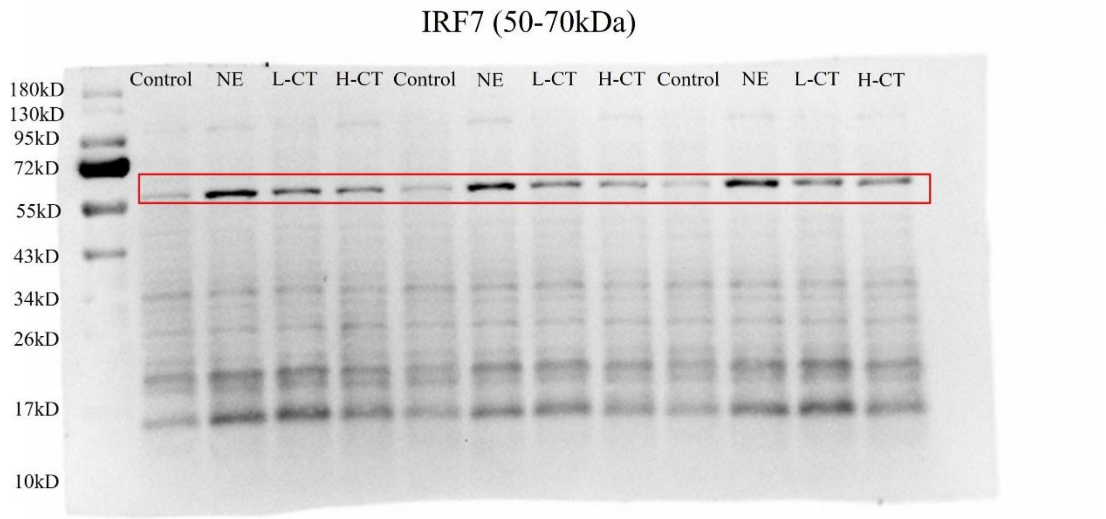

Supplementary Figure S2 (i) The full membrane with molecular weight ladder used for western blot analysis of IRF7 in the jejunum of broilers challenged with necrotic enteritis for day 28 in Figure 6.

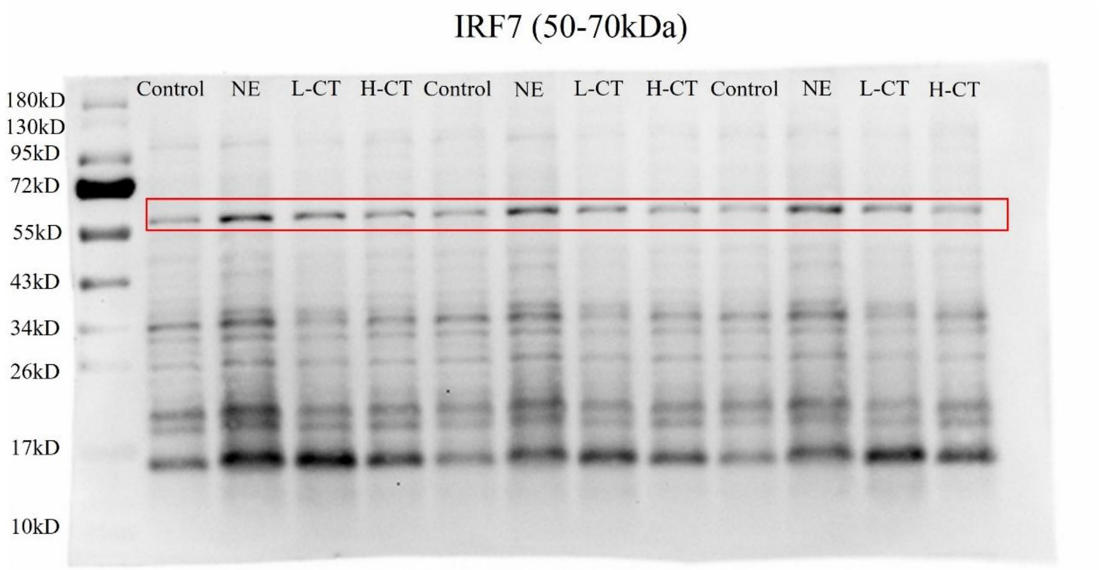

Supplementary Figure S2 (j) The full membrane with molecular weight ladder used for western blot analysis of IRF7 in the jejunum of broilers challenged with necrotic enteritis for day 35 in Figure 6.

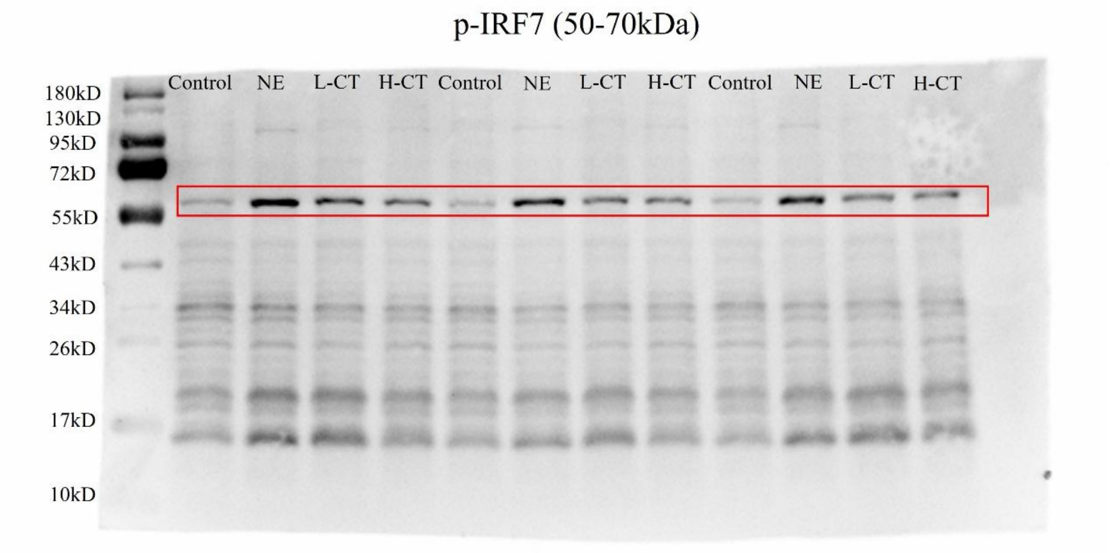

Supplementary Figure S2 (k) The full membrane with molecular weight ladder used for western blot analysis of p-IRF7 in the jejunum of broilers challenged with necrotic enteritis for day 28 in Figure 6.

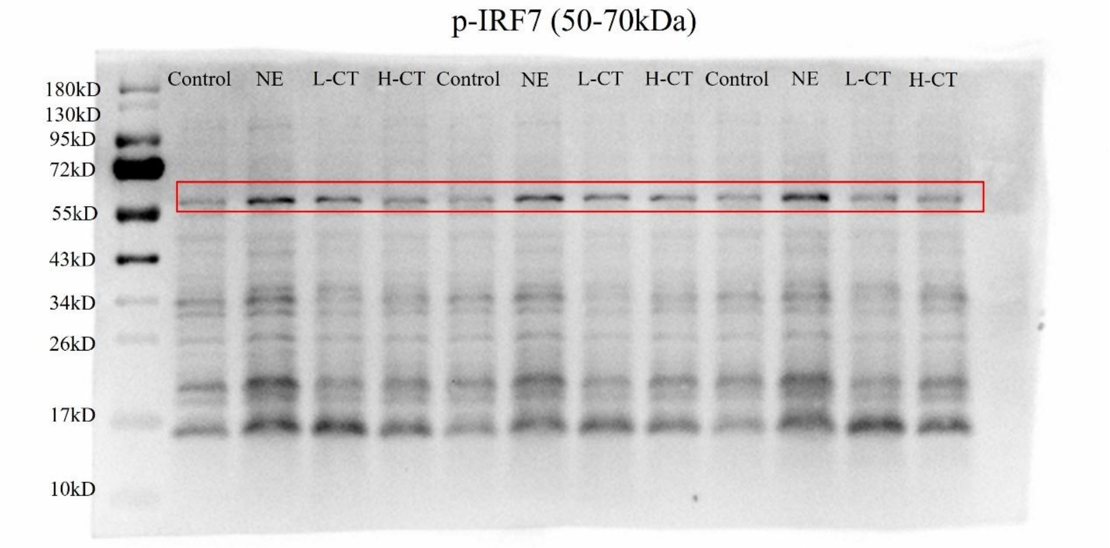

Supplementary Figure S2 (l) The full membrane with molecular weight ladder used for western blot analysis of p-IRF7 in the jejunum of broilers challenged with necrotic enteritis for day 35 in Figure 6.

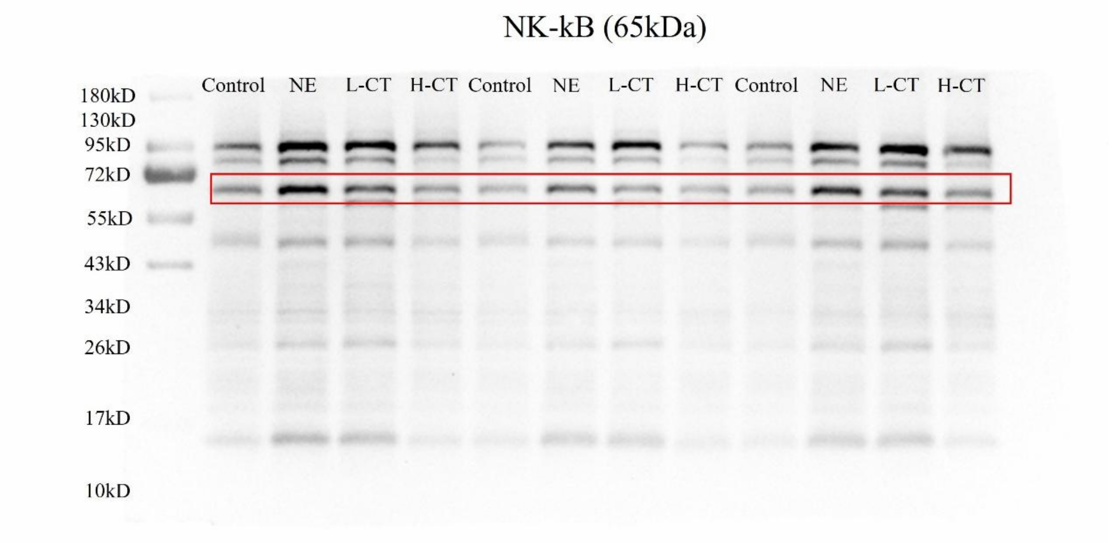

Supplementary Figure S2 (m) The full membrane with molecular weight ladder used for western blot analysis of NF-kB in the jejunum of broilers challenged with necrotic enteritis for day 28 in Figure 6.

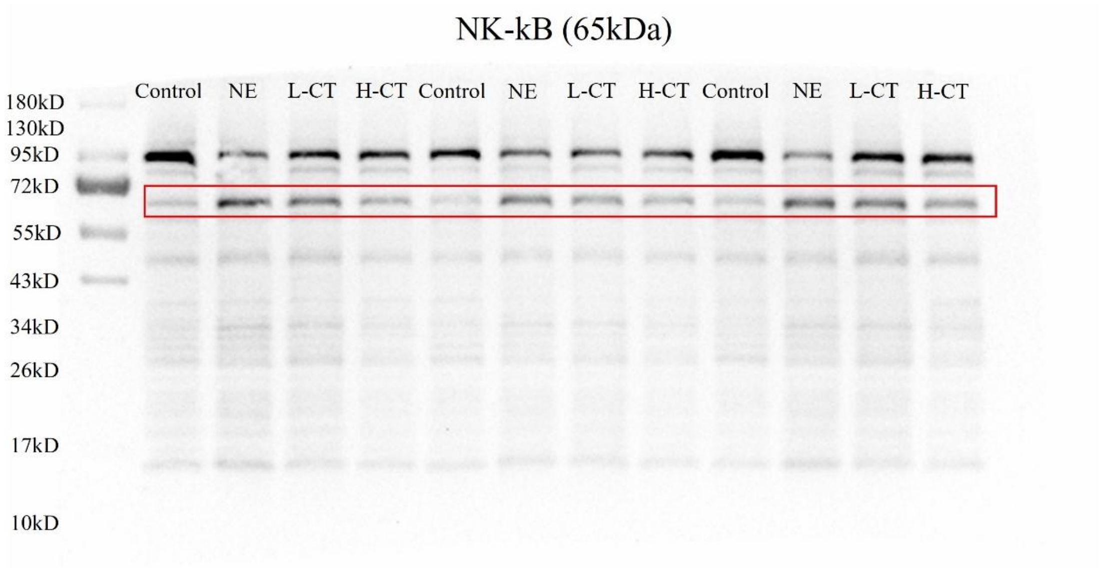

Supplementary Figure S2 (n) The full membrane with molecular weight ladder used for western blot analysis of NF-kB in the jejunum of broilers challenged with necrotic enteritis for day 35 in Figure 6.

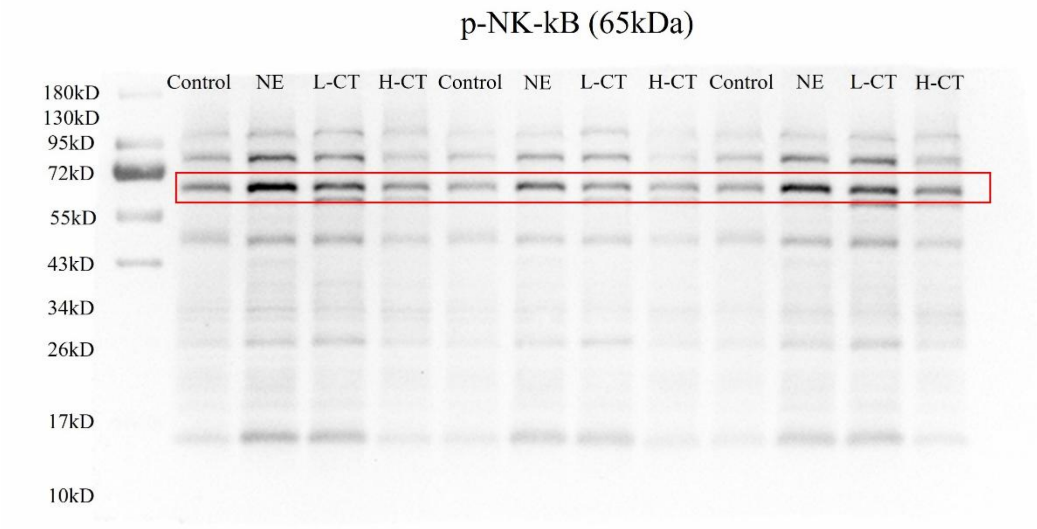

Supplementary Figure S2 (o) The full membrane with molecular weight ladder used for western blot analysis of p-NF-kB in the jejunum of broilers challenged with necrotic enteritis for day 28 in Figure 6.

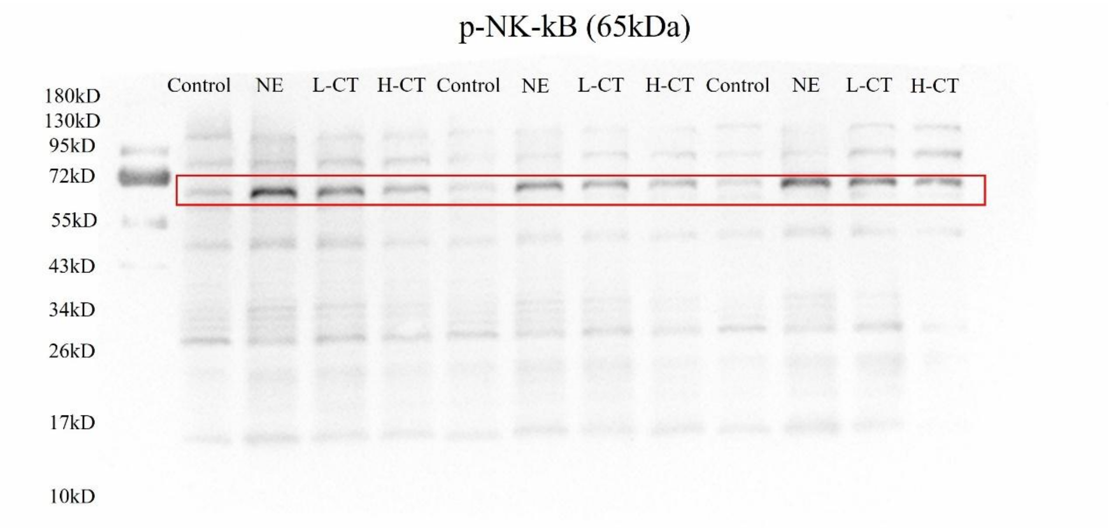

Supplementary Figure S2 (p) The full membrane with molecular weight ladder used for western blot analysis of p-NF-kB in the jejunum of broilers challenged with necrotic enteritis for day 35 in Figure 6.

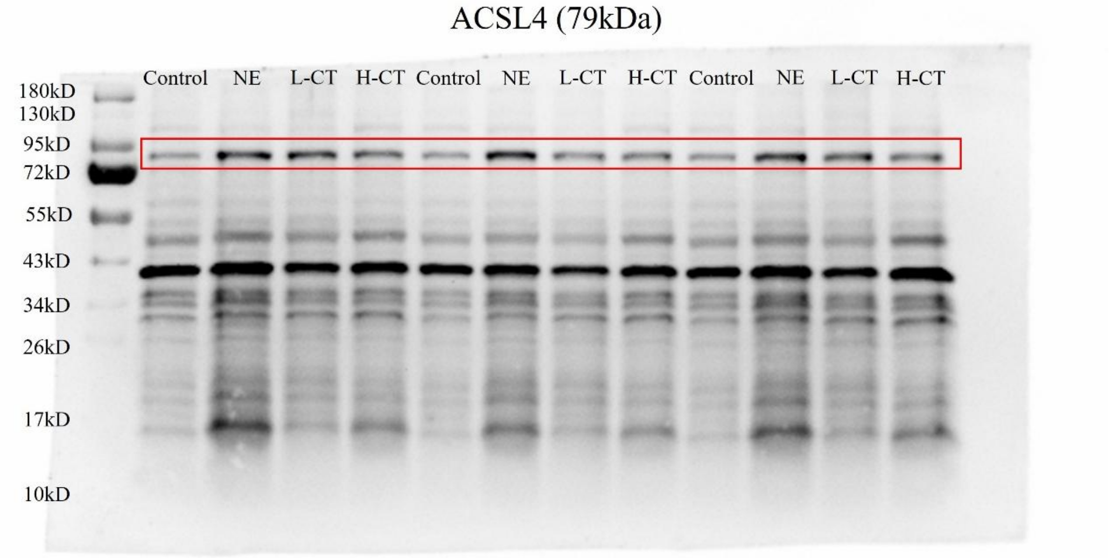

Supplementary Figure S3 (a) The full membrane with molecular weight ladder used for western blot analysis of ACSL4 in the jejunum of broilers challenged with necrotic enteritis for day 28 in Figure 7.

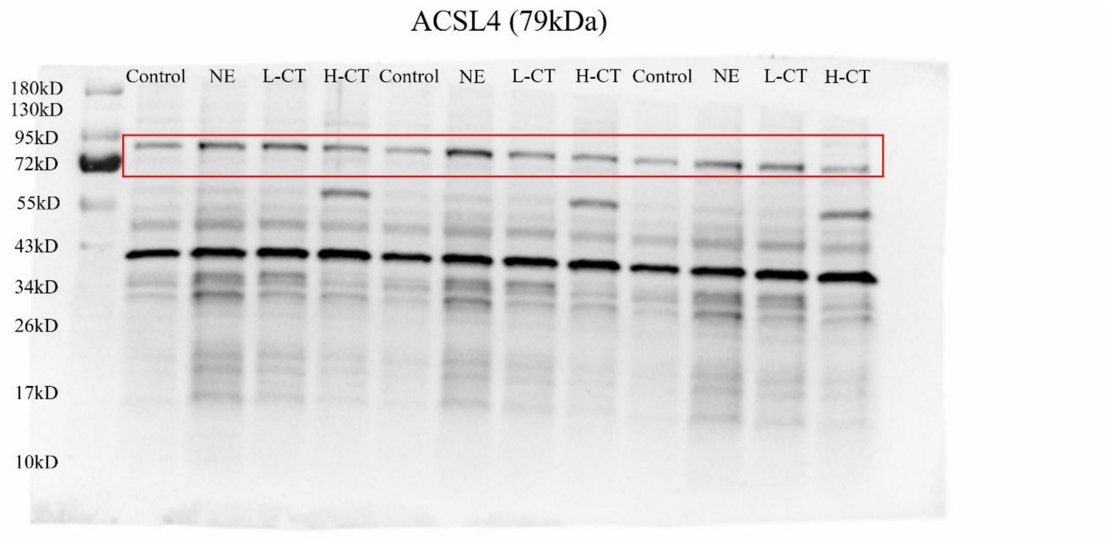

Supplementary Figure S3 (b) The full membrane with molecular weight ladder used for western blot analysis of ACSL4 in the jejunum of broilers challenged with necrotic enteritis for day 35 in Figure 7.

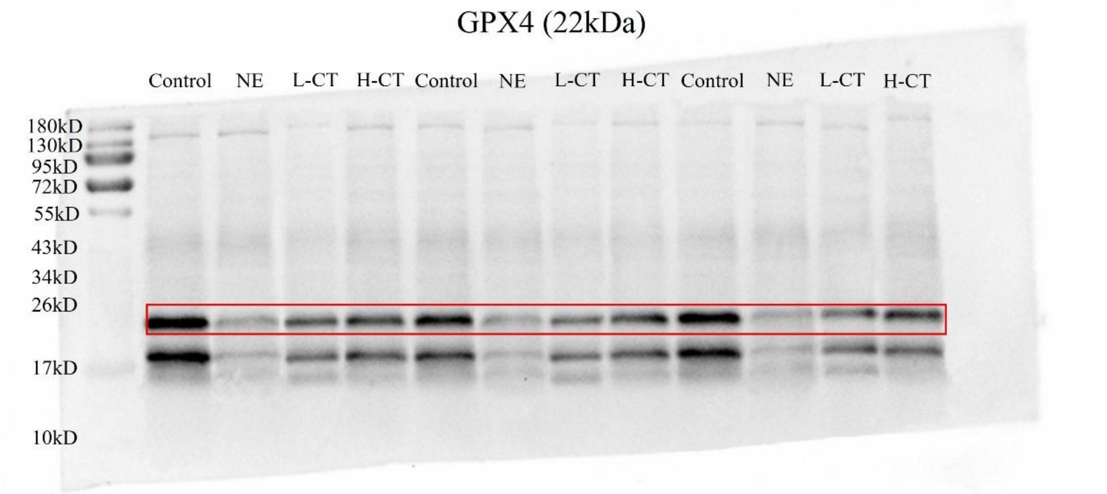

Supplementary Figure S3 (c) The full membrane with molecular weight ladder used for western blot analysis of GPX4 in the jejunum of broilers challenged with necrotic enteritis for day 28 in Figure 7.

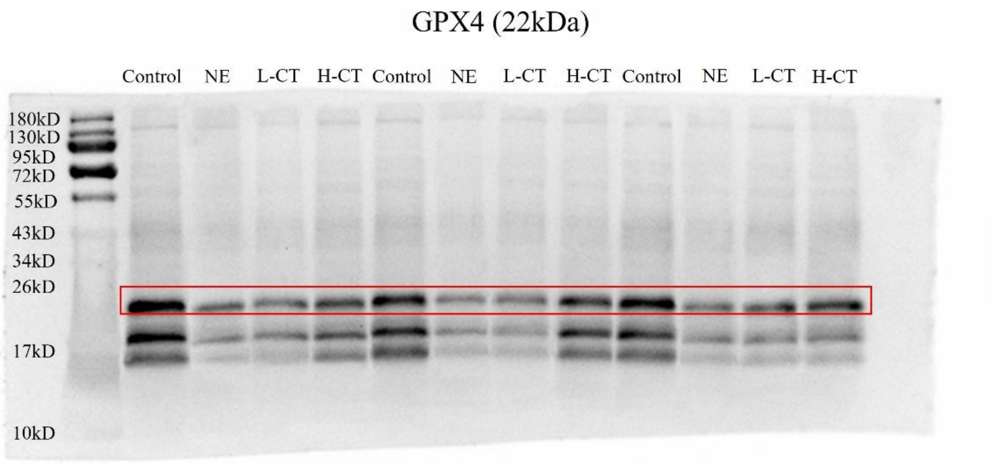

Supplementary Figure S3 (d) The full membrane with molecular weight ladder used for western blot analysis of GPX4 in the jejunum of broilers challenged with necrotic enteritis for day 35 in Figure 7.

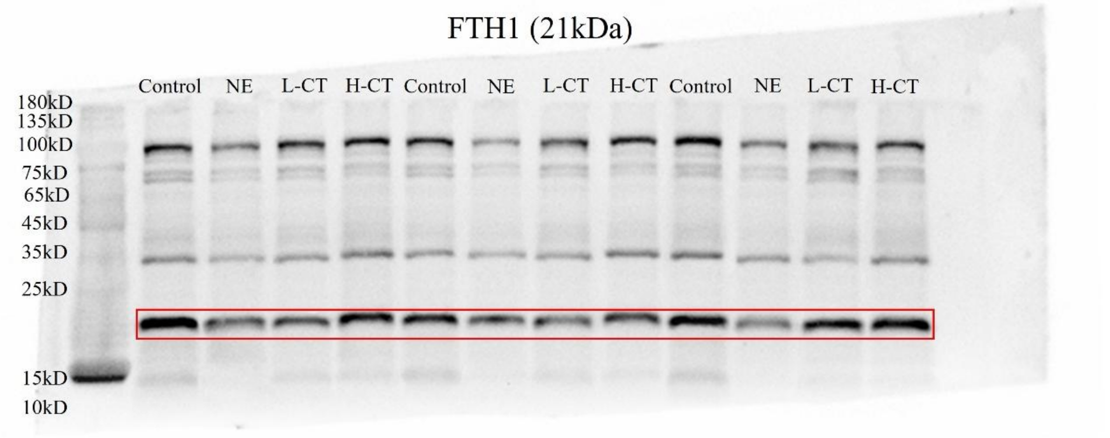

Supplementary Figure S3 (e) The full membrane with molecular weight ladder used for western blot analysis of FTH1 in the jejunum of broilers challenged with necrotic enteritis for day 28 in Figure 7.

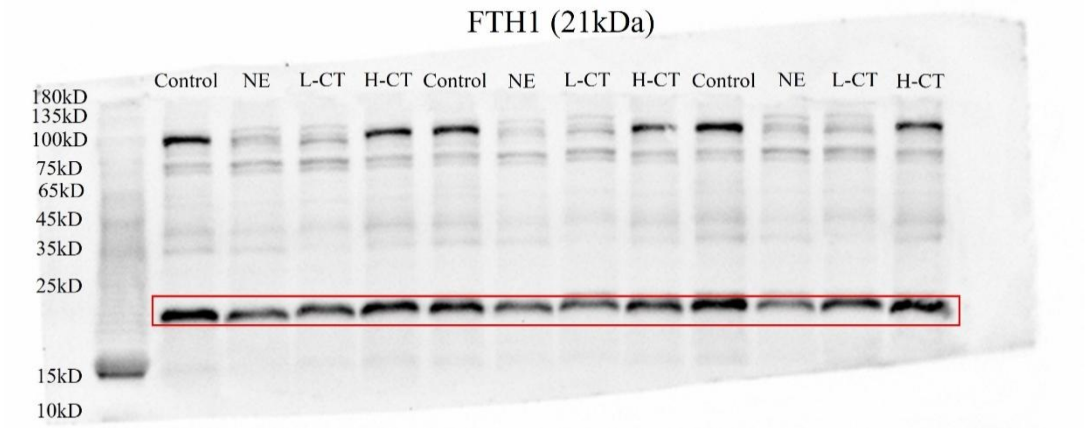

Supplementary Figure S3 (f) The full membrane with molecular weight ladder used for western blot analysis of FTH1 in the jejunum of broilers challenged with necrotic enteritis for day 35 in Figure 7.

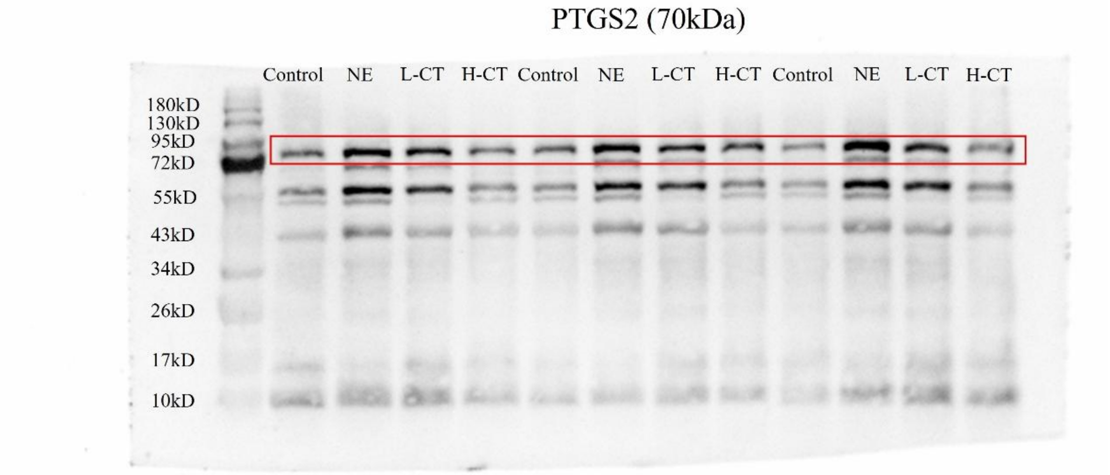

Supplementary Figure S3 (g) The full membrane with molecular weight ladder used for

western blot analysis of PTGS2 in the jejunum of broilers challenged with necrotic enteritis for day 28 in Figure 7.

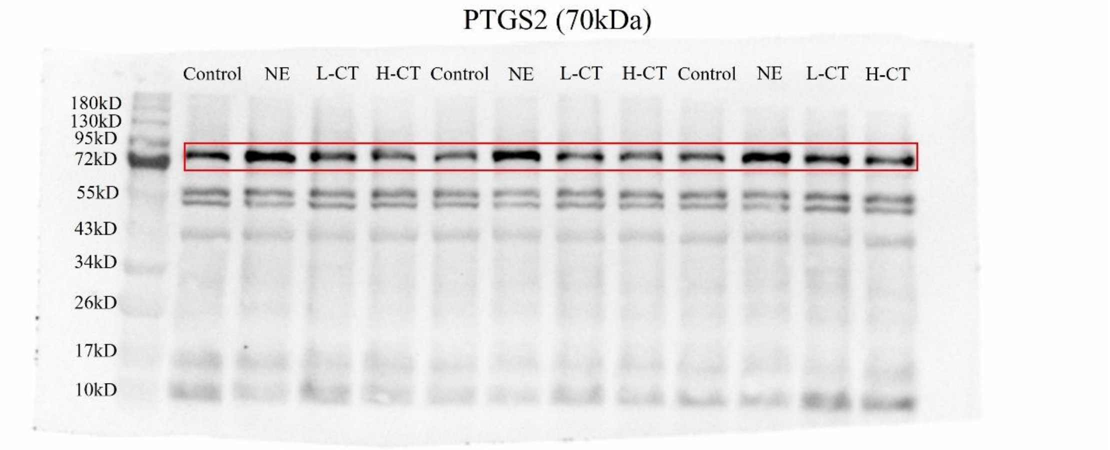

Supplementary Figure S3 (h) The full membrane with molecular weight ladder used for western blot analysis of PTGS2 in the jejunum of broilers challenged with necrotic enteritis for day 35 in Figure 7.

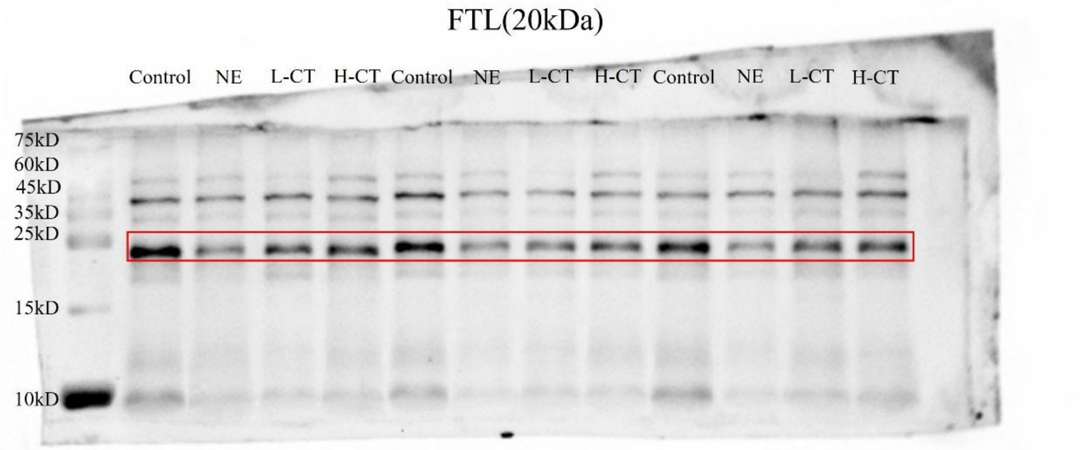

Supplementary Figure S3 (i) The full membrane with molecular weight ladder used for western blot analysis of FTL in the jejunum of broilers challenged with necrotic enteritis for day 28 in Figure 7.

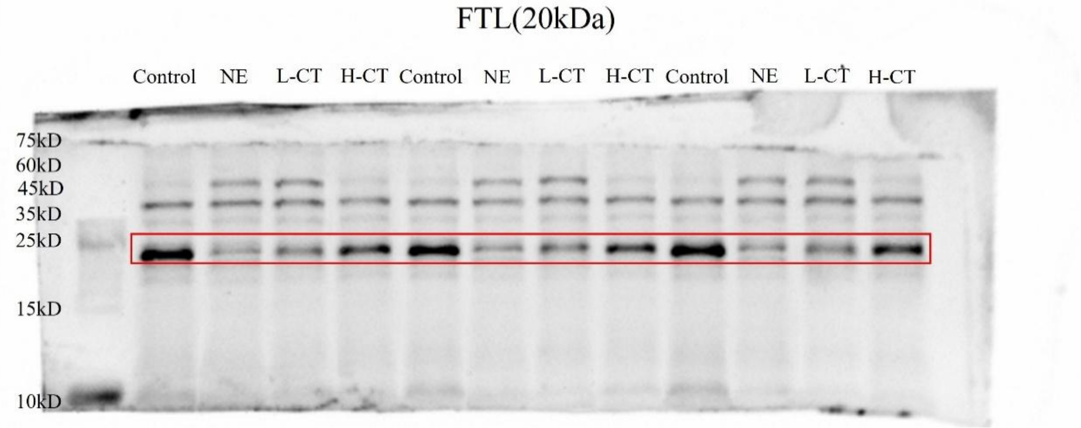

Supplementary Figure S3 (j) The full membrane with molecular weight ladder used for western blot analysis of FTL in the jejunum of broilers challenged with necrotic enteritis for day 35 in Figure 7.

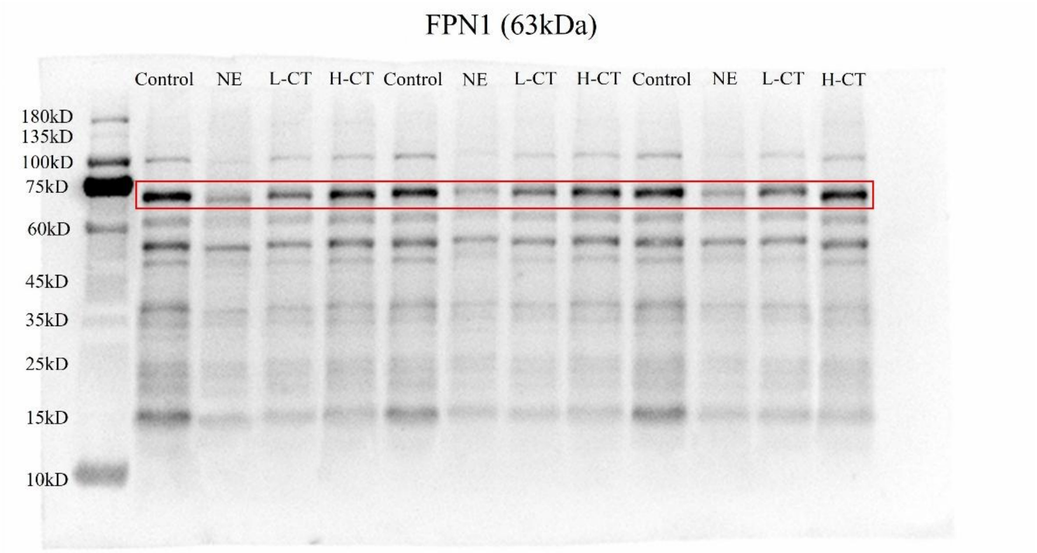

Supplementary Figure S3 (k) The full membrane with molecular weight ladder used for western blot analysis of FPN1 in the jejunum of broilers challenged with necrotic enteritis for day 28 in Figure 7.

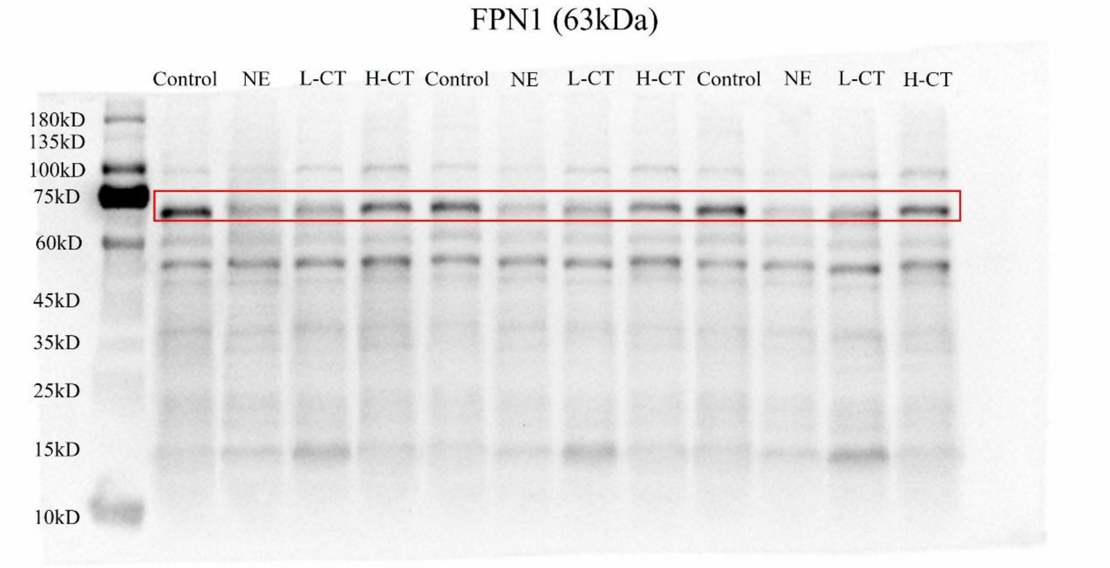

Supplementary Figure S3 (l) The full membrane with molecular weight ladder used for western blot analysis of FPN1 in the jejunum of broilers challenged with necrotic enteritis for day 35 in Figure 7.

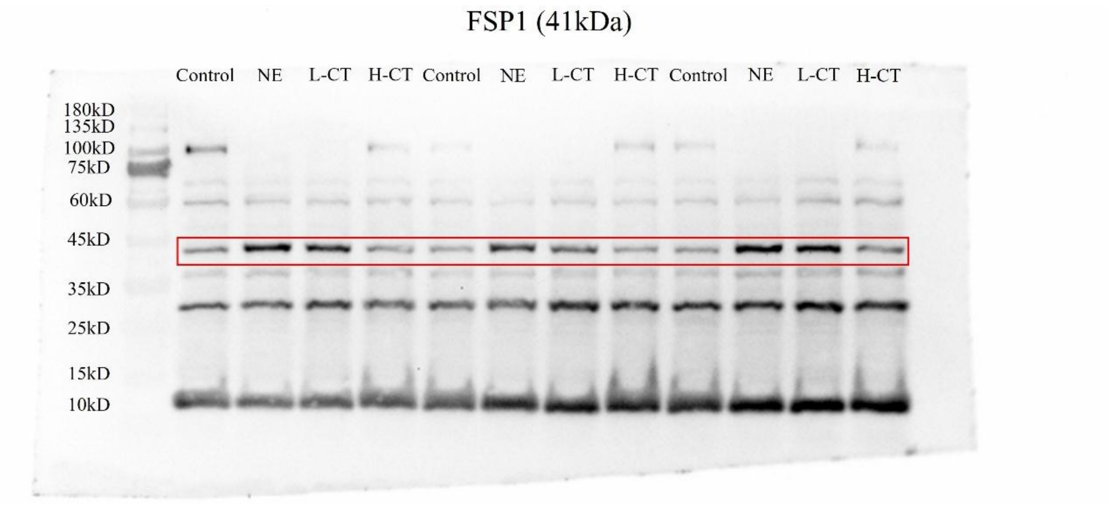

Supplementary Figure S3 (m) The full membrane with molecular weight ladder used for western blot analysis of FSP1 in the jejunum of broilers challenged with necrotic enteritis for day 28 in Figure 7.

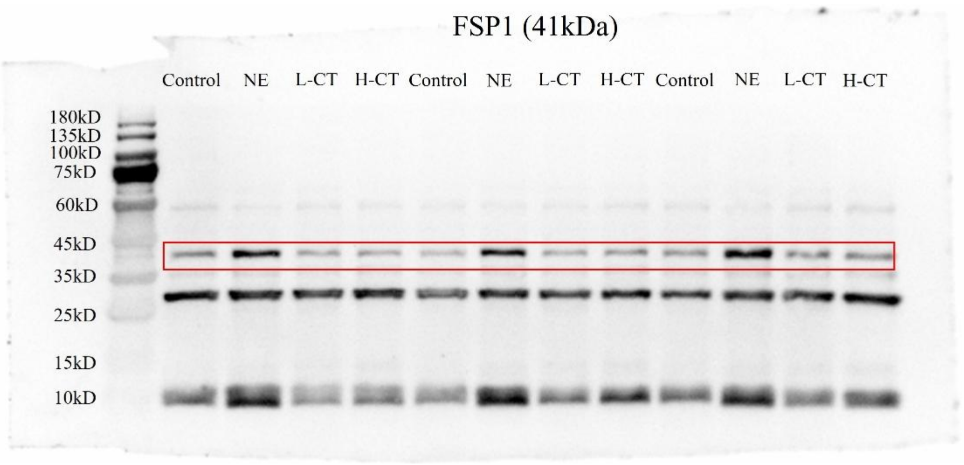

Supplementary Figure S3 (n) The full membrane with molecular weight ladder used for western blot analysis of FSP1 in the jejunum of broilers challenged with necrotic enteritis for day 35 in Figure 7.

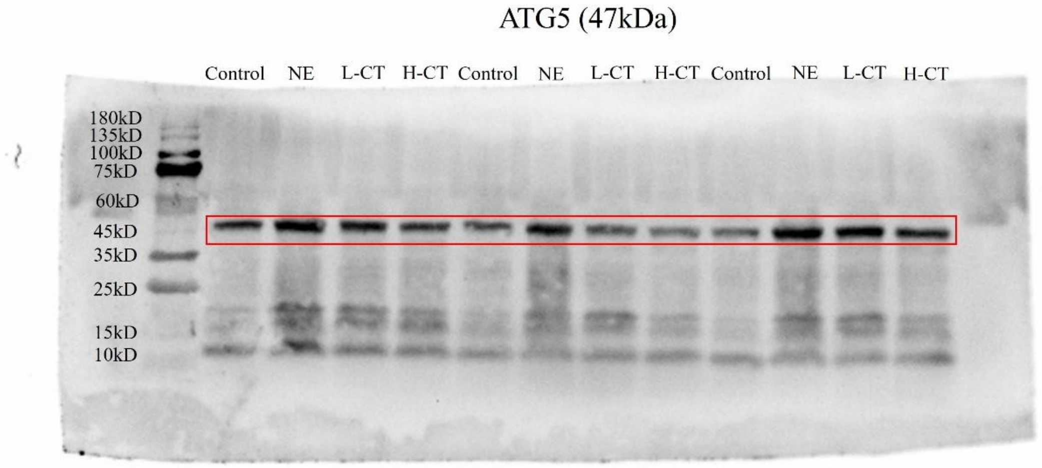

Supplementary Figure S4 (a) The full membrane with molecular weight ladder used for

western blot analysis of ATG5 in the jejunum of broilers challenged with necrotic enteritis for day 28 in Figure 8.

ATG5 (47kDa)

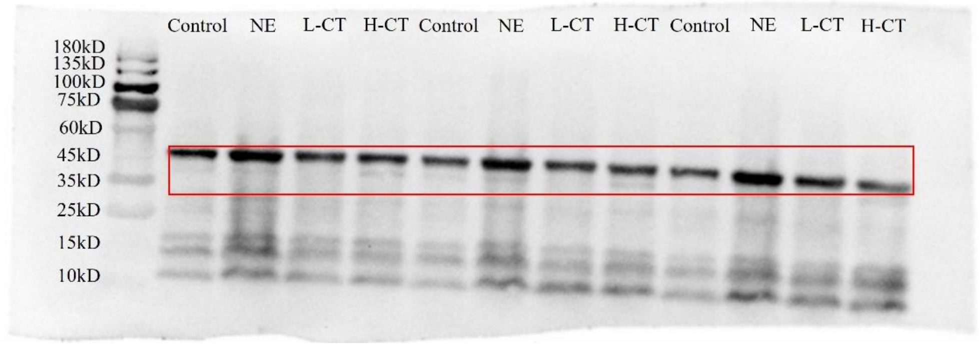

Supplementary Figure S4 (b) The full membrane with molecular weight ladder used for western blot analysis of ATG5 in the jejunum of broilers challenged with necrotic enteritis for day 35 in Figure 8.

WIPI2 (49kDa)

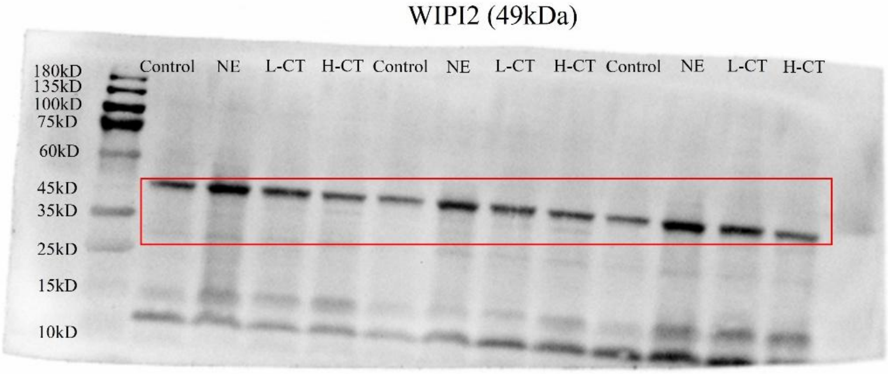

Supplementary Figure S4 (c) The full membrane with molecular weight ladder used for western blot analysis of WIPI2 in the jejunum of broilers challenged with necrotic enteritis for day 28 in Figure 8.

WIPI2 (49kDa)

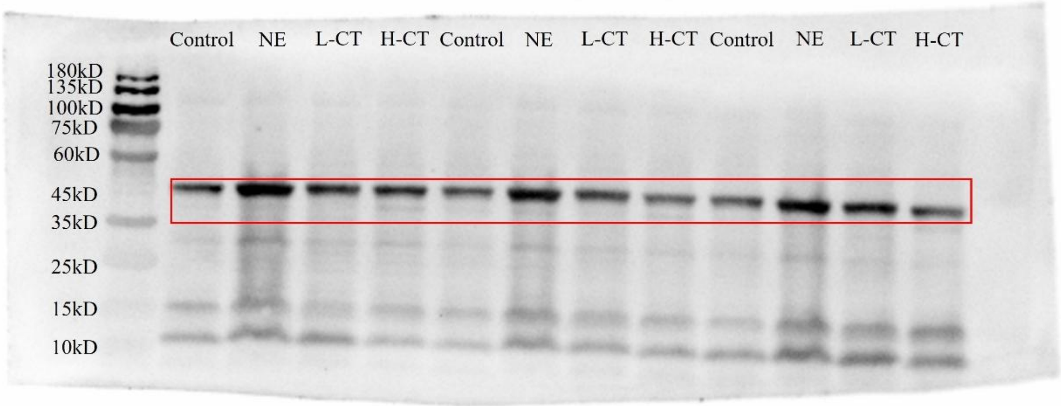

Supplementary Figure S4 (d) The full membrane with molecular weight ladder used for western blot analysis of WIPI2 in the jejunum of broilers challenged with necrotic enteritis for day 35 in Figure 8.

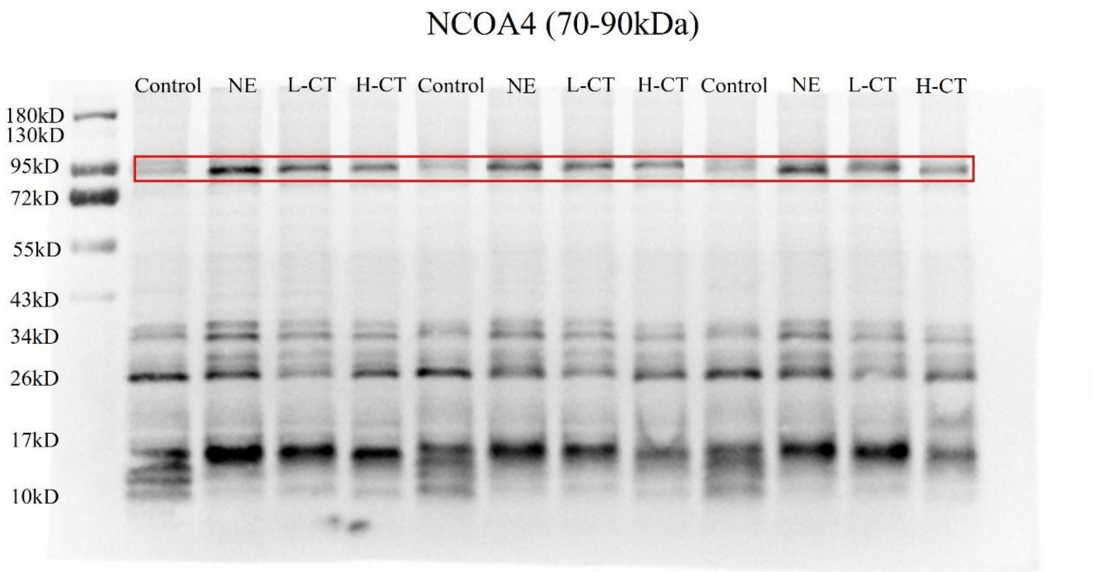

Supplementary Figure S4 (e) The full membrane with molecular weight ladder used for western blot analysis of NCOA4 in the jejunum of broilers challenged with necrotic enteritis for day 28 in Figure 8.

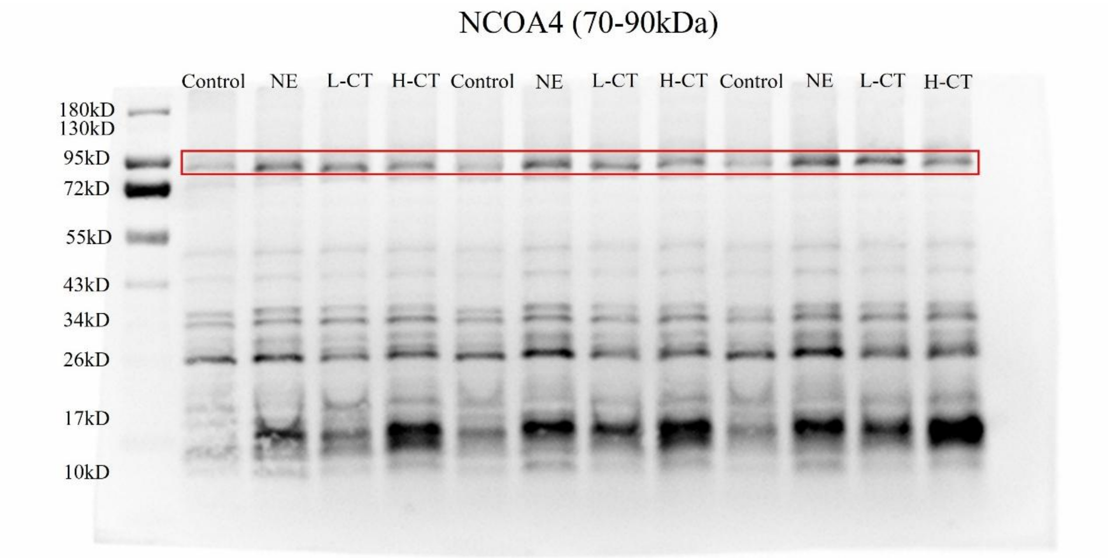

Supplementary Figure S4 (f) The full membrane with molecular weight ladder used for western blot analysis of NCOA4 in the jejunum of broilers challenged with necrotic enteritis for day 35 in Figure 8.
